# Supplementary material for: Density Functional Method Study on the Cooperativity of Intermolecular H-bonding and π-π+ Stacking Interactions in Thymine-[Cnmim]Br (n = 2, 4, 6, 8, 10) Microhydrates
Source: Molecules. 2022 Sep 22;27(19):6242. doi: 10.3390/molecules27196242 (PMC9572290; doi:10.3390/molecules27196242)
Supplement: Supplementary file 1 [file molecules-27-06242-s001.zip › Electronic Supplementary Information-Figure S1-S8.pdf]

Electronic Supplementary Information (ESI)

Density Functional Method Study on the Cooperativity of Intermolecular H-bonding and π-π+ Stacking Interactions in Thymine-[$C_n$mim]Br (n = 2, 4, 6, 8, 10) Microhydrates

Yanni Wang[1], Chaowu Dai[1], Wei Huang[1], Tingting Ni[2], Jianping Cao[2], Jiangmei Pang[2], Huining Wei[2] and Chaojie Wang[2*]

[1]The Third Affiliated Hospital of Wenzhou Medical University; the Pharmacy Department of Ruian People's Hospital, Wenzhou 325000, Zhejiang, China.

[2]School of Pharmaceutical Sciences, Wenzhou Medical University, Wenzhou 325035, Zhejiang, China.

[*]Correspondence: chjwang@wmu.edu.cn

Contents

Figure S1. Optimized molecular structures of micro-hydrated T-*w*H_2O (*w* = 1, 2, 3, 4, 5) at the M06-2X/6-311++G(2*d*, *p*)/PCM/water level. The ZPVE-corrected stability decreases from left to right (in kJ/mol). 2

Figure S2. Optimized molecular structures of mono-anionic T-*w*H_2O-Br^- (*w* = 1, 2, 3) at the M06-2X/6-311++G(2*d*, *p*)/PCM/water level. The ZPVE-corrected stability decreases from left to right (in kJ/mol). 5

Figure S3. 11 of the mono-cationic structures of thymine with five imidazolium cations (T-[$C_n$mim]$^+$, n = 2, 4, 6, 8, 10) calculated at the M06-2X/6-311++G(2*d*, *p*)/PCM/water level of theory. The ZPVE-corrected stability decreases from top to bottom for each configuration (in kJ/mol). 15

Figure S4. 12 of the di-ionic structures of thymine with five imidazolium ILs (T-[$C_n$mim]$^+$-Br^- , n = 2, 4, 6, 8, 10) calculated at the M06-2X/6-311++G(2*d*, *p*)/PCM/water level of theory. The ZPVE-corrected stability decreases from top to bottom for each configuration (in kJ/mol). 25

Figure S5. 3 of the tri-ionic structures of thymine with five imidazolium ILs (T-[$C_n$mim]$^+$-2Br^-, n = 2, 4, 6, 8, 10) based on the stacking, perpendicular, and coplanar configurations and calculated at the M06-2X/6-311++G(2*d*, *p*)/PCM/water level of theory. The ZPVE-corrected stability decreases from left to right (in kJ/mol). 28

Figure S6. 3 of the tetra-ionic structures of thymine with five imidazolium ILs (T-2[$C_n$mim]$^+$-2Br^-, n = 2, 4, 6, 8, 10) based on the stacking, perpendicular, and coplanar configurations and calculated at the M06-2X/6-311++G(2*d*, *p*)/PCM/water level of theory. The ZPVE-corrected stability decreases from left to right (in kJ/mol). 32

Figure S7. Optimized molecular structures of micro-hydrated T-*w*H_2O-[$C_n$mim]$^+$-Br^- (*w* = 1, 2 and n = 2, 4, 6, 8, 10) at the M06-2X/6-311++G(2*d*, *p*)/PCM/water level. The ZPVE-corrected stability decreases from left to right (in kJ/mol). 37

Figure S8. The isosurface maps of the frontier molecular orbitals for the isolated T, micro-hydrated T-4H_2O-Br^--1, T-3H_2O-[$C_n$mim]$^+$-Br^--1, T-2H_2O-[$C_n$mim]$^+$-2Br^--1, and T-1H_2O-2[$C_n$mim]$^+$-2Br^--1 species calculated at the TD-M06-2X/6-311++G(2*d*, *p*)/PCM/water level (isovalues = 0.01 a.u.). 43

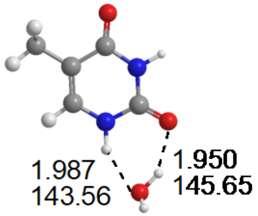

T-1H$_2$O-1 (0.00)

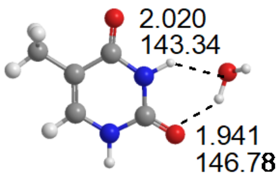

T-1H$_2$O-2 (0.65)

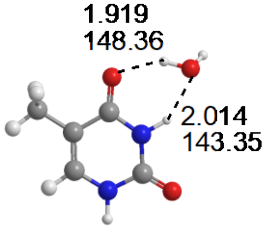

T-1H$_2$O-3 (0.80)

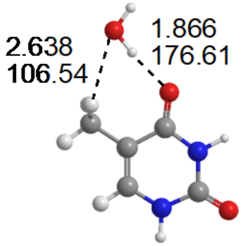

T-1H$_2$O-4 (4.92)

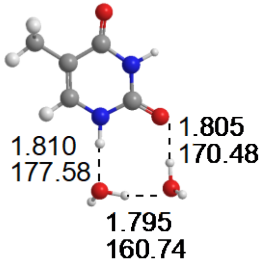

T-2H$_2$O-1 (0.00)

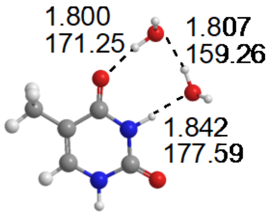

T-2H$_2$O-2 (1.71)

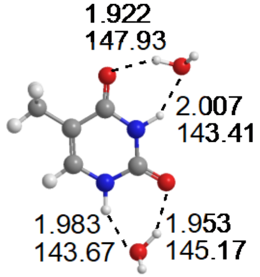

T-2H$_2$O-3 (6.50)

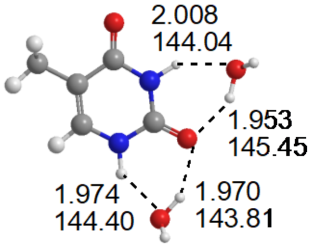

T-2H$_2$O-4 (6.80)

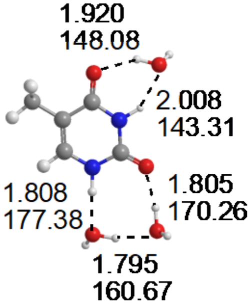

T-3H$_2$O-1 (0.00)

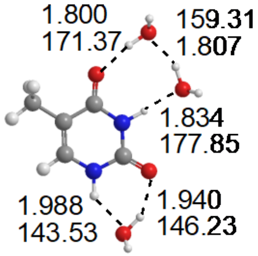

T-3H$_2$O-2 (0.80)

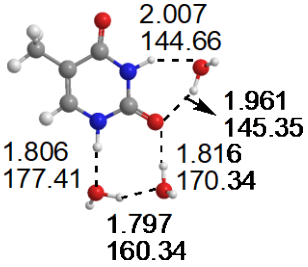

T-3H$_2$O-3 (1.25)

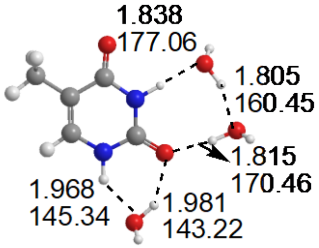

T-3H$_2$O-4 (2.10)

Continued

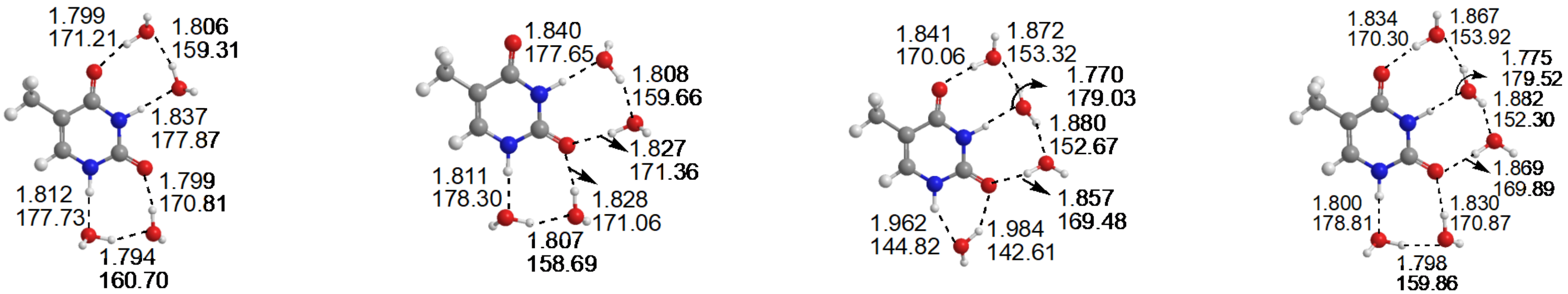

T-4H_2O-1 (0.00) T-4H_2O-2 (2.21) T-4H_2O-3 (4.07) T-5H_2O-1 (-97.39 kJ/mol)

Figure S1. Optimized molecular structures of micro-hydrated T-*w*H_2O (*w* = 1, 2, 3, 4, 5) at the M06-2X/6-311++G(2*d*, *p*)/PCM/water level. The ZPVE-corrected stability decreases from left to right (in kJ/mol), but T-5H2O-1 system the binding energy listed.

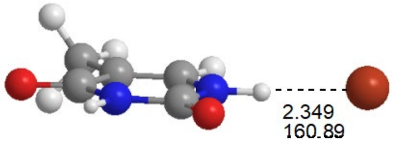

T-Br^{-}-1 (0.00)

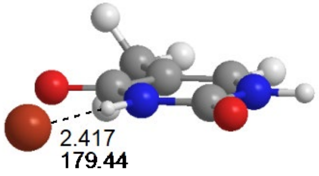

T-Br^{-}-2 (5.71)

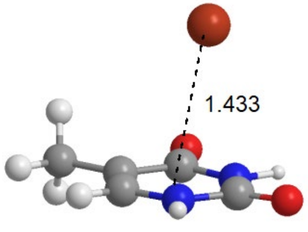

T-Br^{-}-3 (15.75)

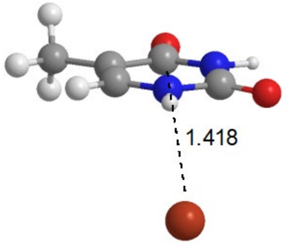

T-Br^{-}-4 (15.76)

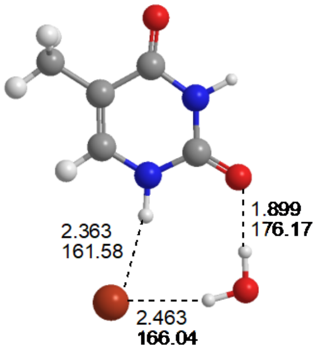

T-1H_2O-Br^{-}-1 (0.00)

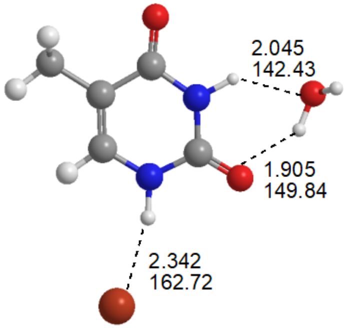

T-1H_2O-Br^{-}-2 (5.58)

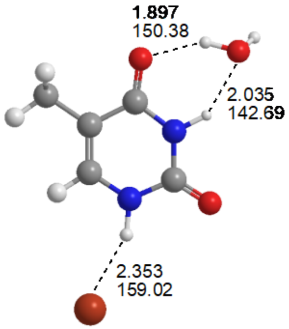

T-1H_2O-Br^{-}-3 (5.56)

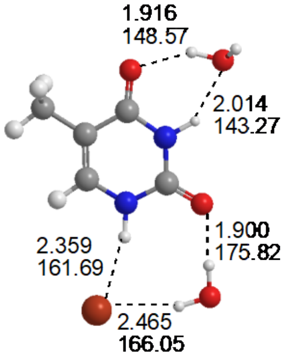

T-2H_2O-Br^{-}-1 (0.00)

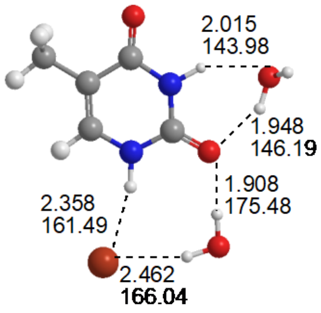

T-2H_2O-Br^{-}-2 (0.66)

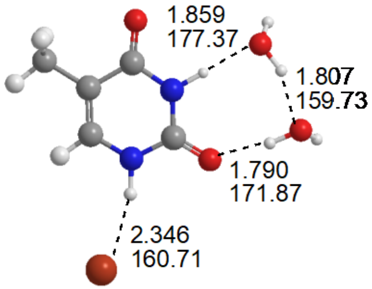

T-2H_2O-Br^{-}-3 (0.79)

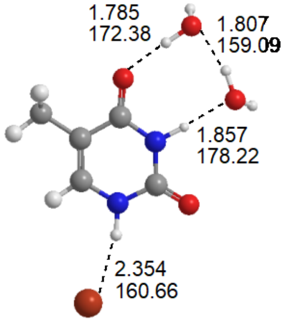

T-2H_2O-Br^{-}-4 (1.17)

Continued

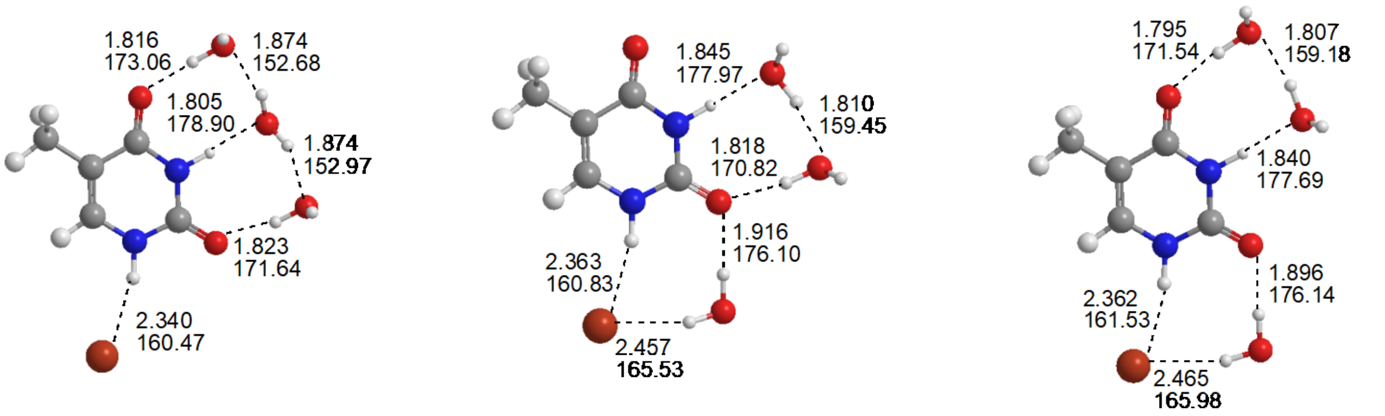

T-3H_2O-Br^--1 (0.00) T-3H_2O-Br^--2 (0.25) T-3H_2O-Br^--3 (1.25)

Figure S2. Optimized molecular structures of mono-anionic T-$w$$H_2$O-$Br^-$ (w = 1, 2, 3) at the M06-2X/6-311++G(2*d*, *p*)/PCM/water level. The ZPVE-corrected stability decreases from left to right (in kJ/mol).

Stacking (S)

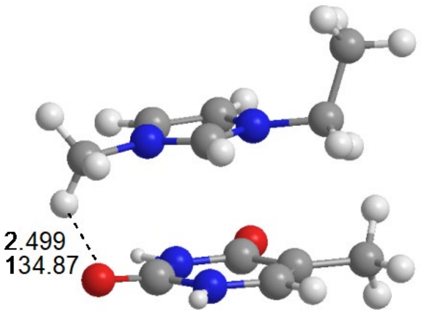

T-[C_2mim]$^+$-1 (0.00)

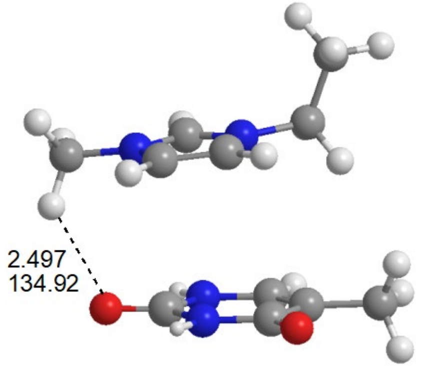

T-[C_2mim]$^+$-2 (0.10)

Perpendicular (P)

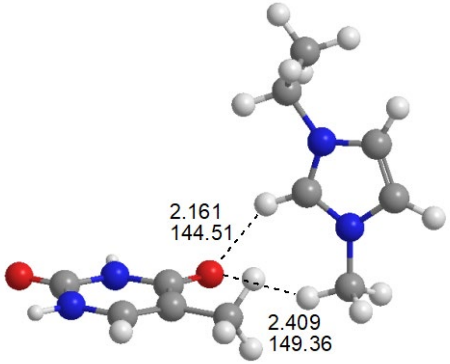

T-[C_2mim]$^+$-5 (0.00)

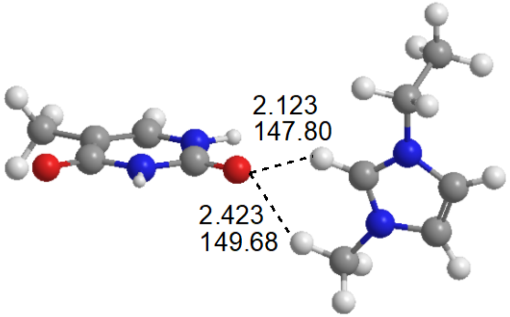

T-[C_2mim]$^+$-6 (3.07)

Coplanar (H)

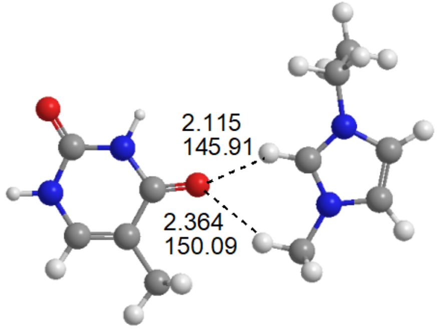

T-[C_2mim]$^+$-9 (0.00)

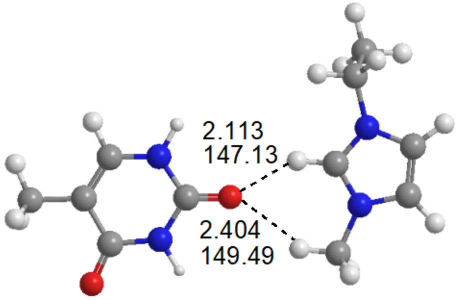

T-[C_2mim]$^+$-10 (1.79)

Continued

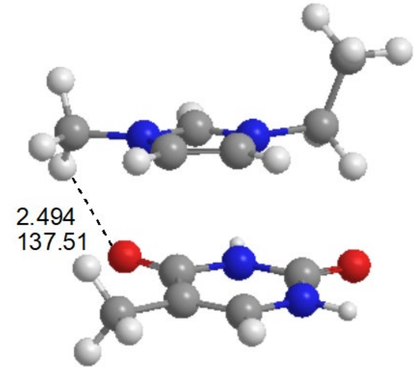

T-$[C_2mim]^+$-3 (0.23)

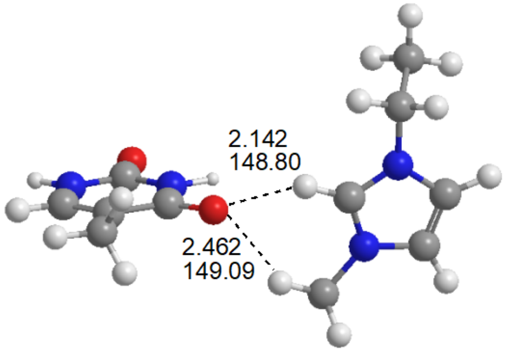

T-$[C_2mim]^+$-7 (3.10)

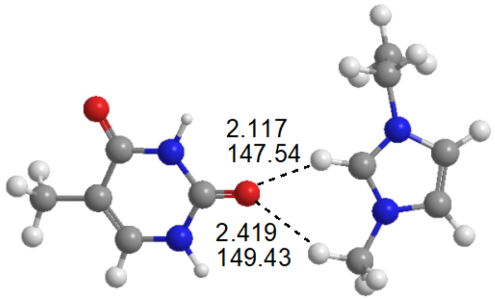

T-$[C_2mim]^+$-11 (2.15)

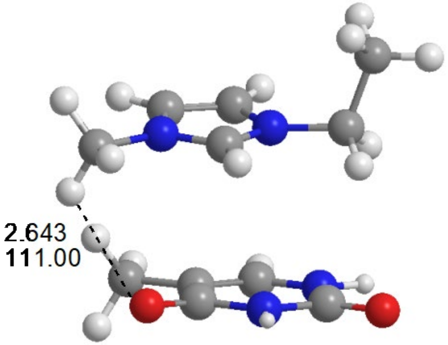

T-$[C_2mim]^+$-4 (0.24)

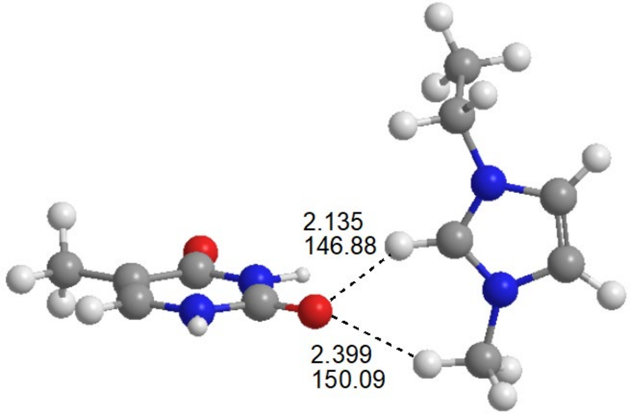

T-$[C_2mim]^+$-8 (3.40)

Stacking (S)

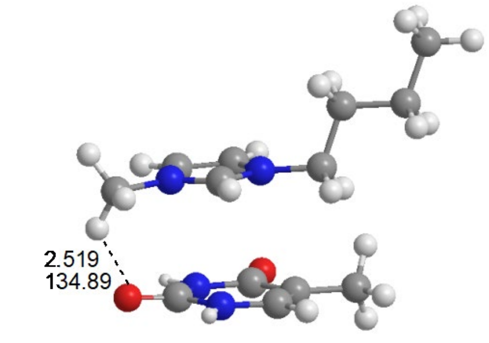

T-$[C_4mim]^+$-1 (0.00)

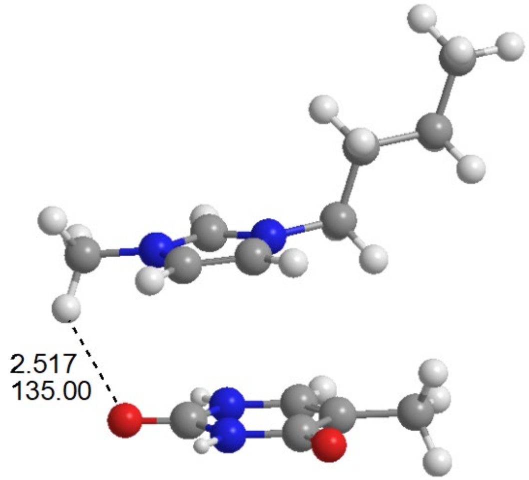

T-$[C_4mim]^+$-2 (0.01)

Perpendicular (P)

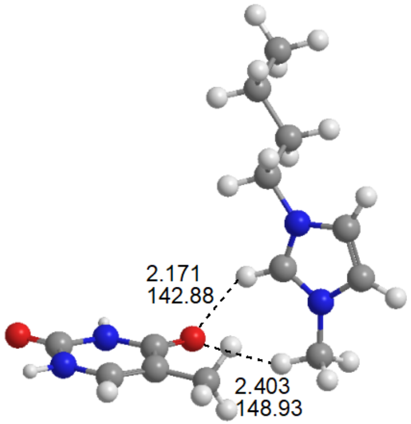

T-$[C_4mim]^+$-5 (0.00)

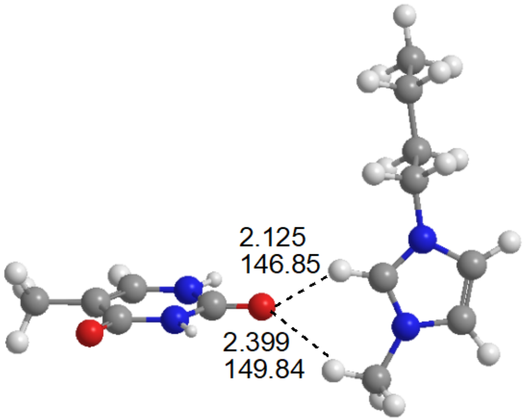

T-$[C_4mim]^+$-6 (2.13)

Coplanar (H)

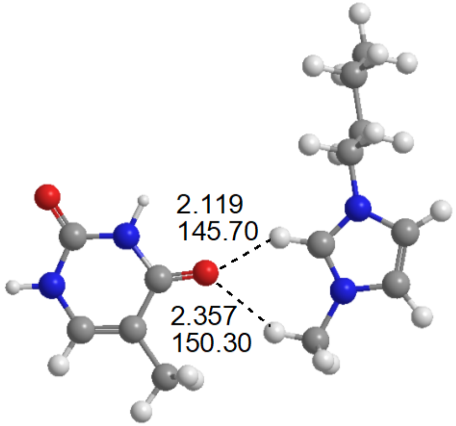

T-$[C_4mim]^+$-9 (0.00)

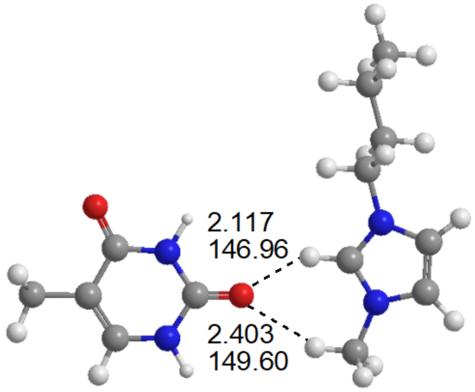

T-$[C_4mim]^+$-10 (2.13)

Continued

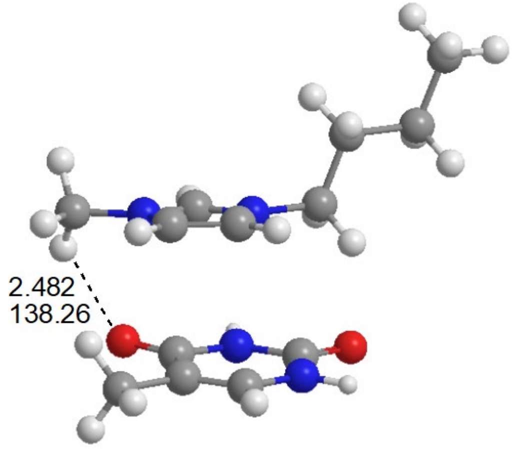

T-[C_4mim]$^+$-3 (0.02)

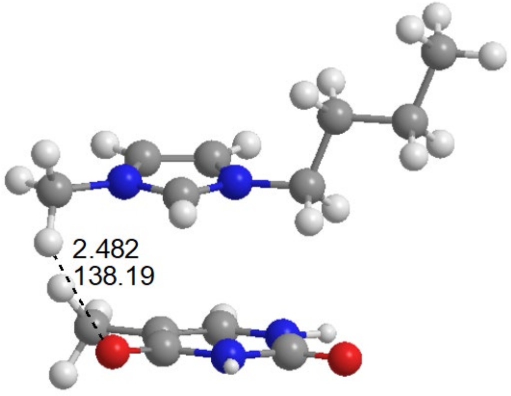

T-[C_4mim]$^+$-4 (0.04)

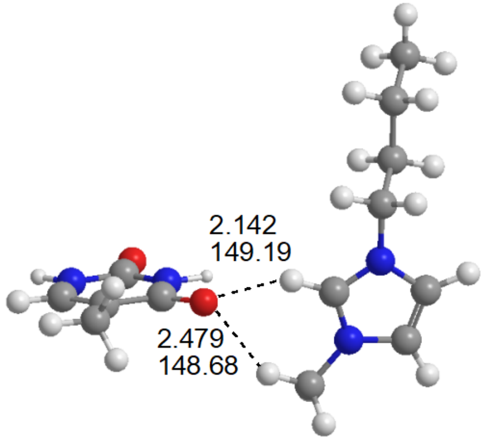

T-[C_4mim]$^+$-7 (2.61)

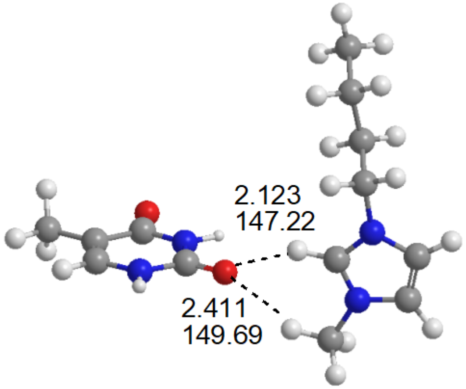

T-[C_4mim]$^+$-8 (2.91)

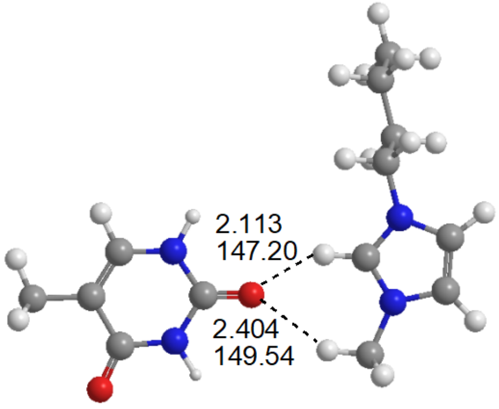

T-[C_4mim]$^+$-11 (2.40)

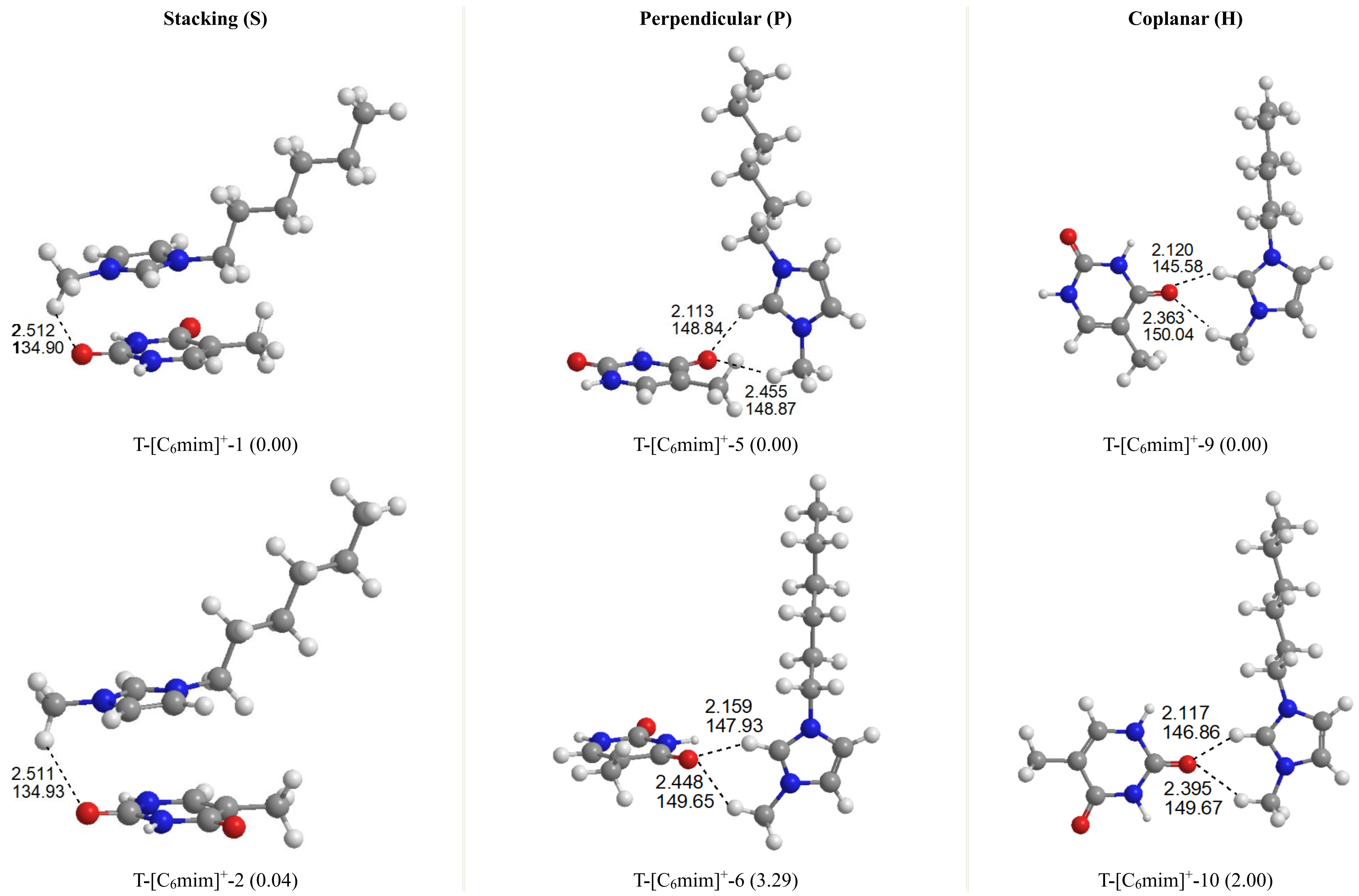

Stacking (S)
Perpendicular (P)
Coplanar (H)
2.512
134.90
T-[C6mim]+-1 (0.00)
2.113
148.84
2.455
148.87
T-[C6mim]+-5 (0.00)
2.120
145.58
2.363
150.04
T-[C6mim]+-9 (0.00)
2.511
134.93
T-[C6mim]+-2 (0.04)
2.159
147.93
2.448
149.65
T-[C6mim]+-6 (3.29)
2.117
146.86
2.395
149.67
T-[C6mim]+-10 (2.00)

Continued

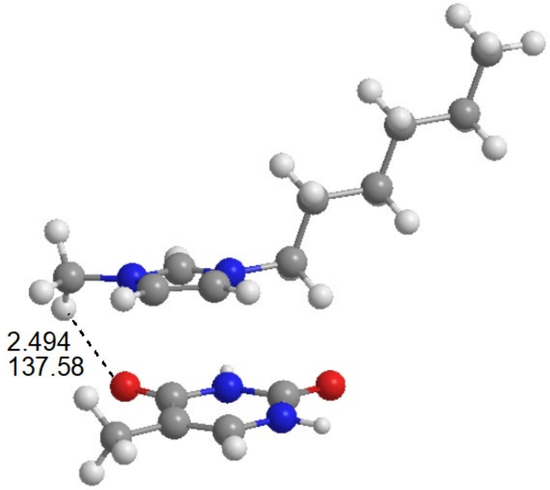

T-[C_6mim]$^+$-3 (0.27)

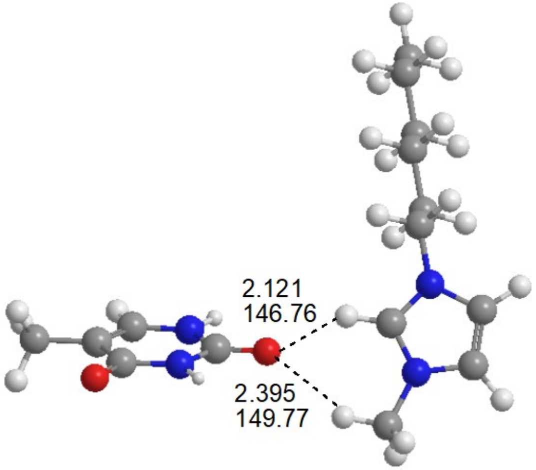

T-[C_6mim]$^+$-7 (3.52)

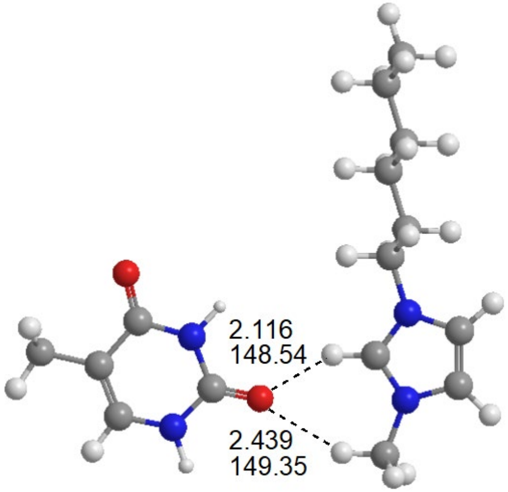

T-[C_6mim]$^+$-11 (2.37)

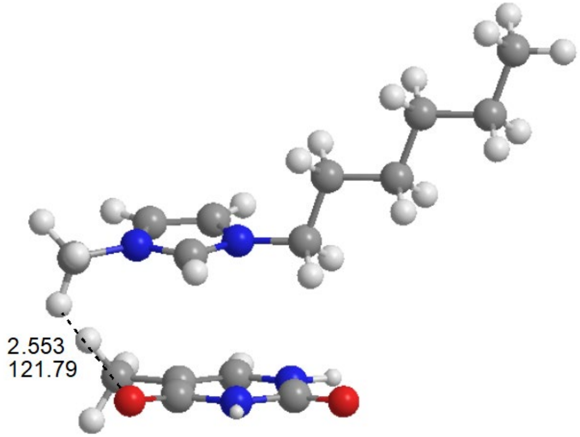

T-[C_6mim]$^+$-4 (0.73)

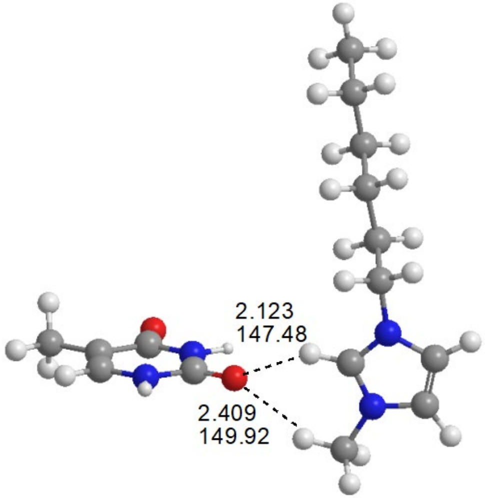

T-[C_6mim]$^+$-8 (3.77)

Stacking (S) | **Perpendicular (P)** | **Coplanar (H)**

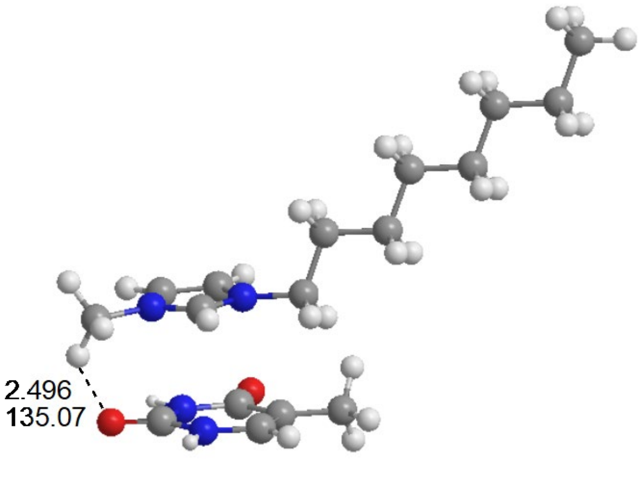

T-[C_8mim]$^+$-1 (0.00)

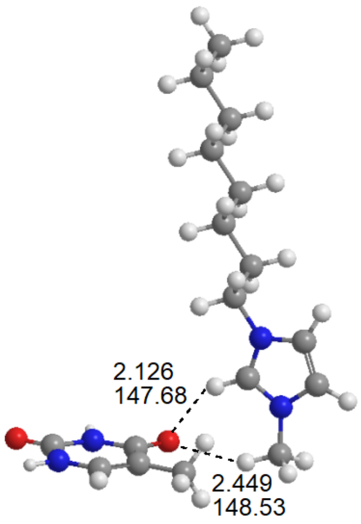

T-[C_8mim]$^+$-5 (0.00)

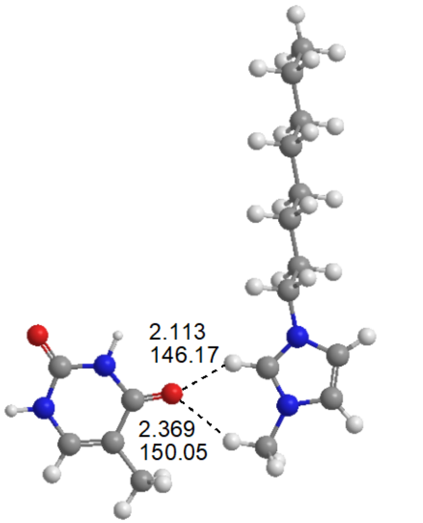

T-[C_8mim]$^+$-9 (0.00)

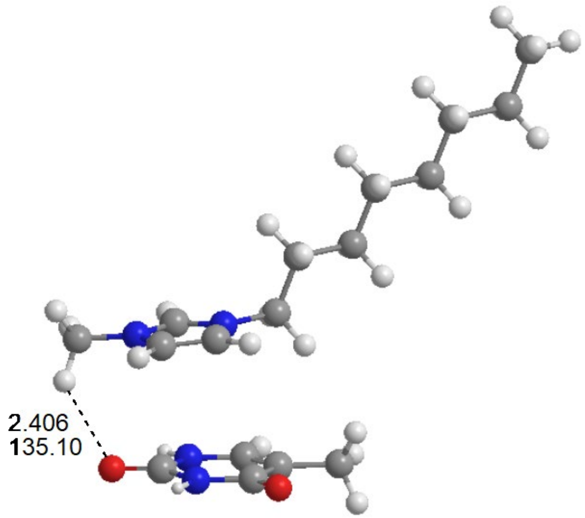

T-[C_8mim]$^+$-2 (0.01)

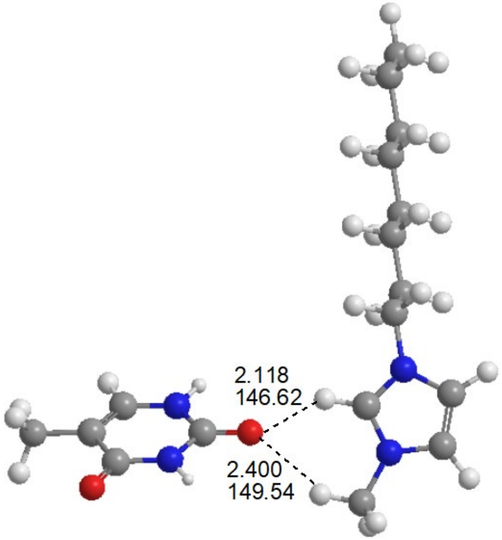

T-[C_8mim]$^+$-6 (3.44)

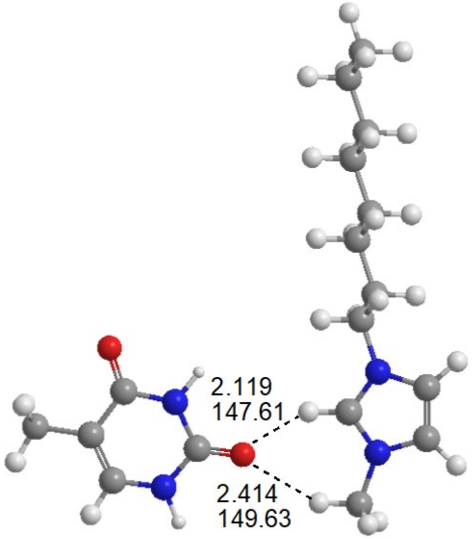

T-[C_8mim]$^+$-10 (2.17)

Continued

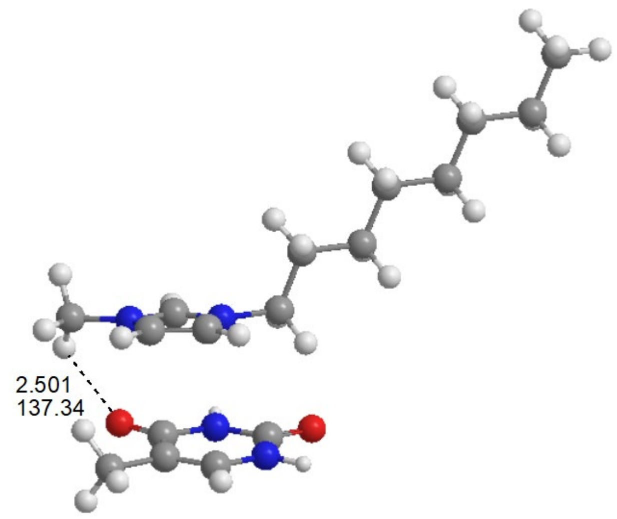

T-[C_8mim]$^+$-3 (0.77)

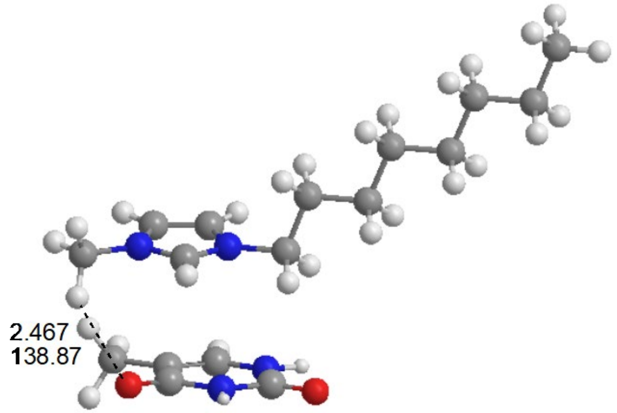

T-[C_8mim]$^+$-4 (0.85)

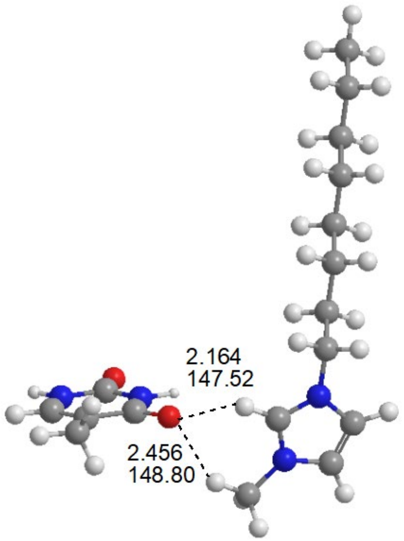

T-[C_8mim]$^+$-7 (3.80)

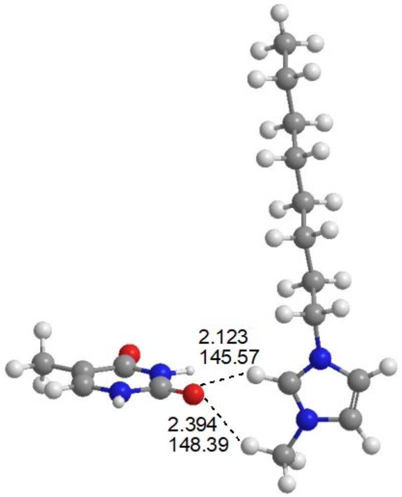

T-[C_8mim]$^+$-8 (4.07)

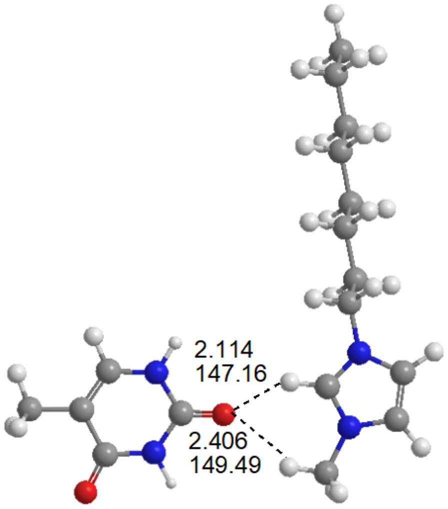

T-[C_8mim]$^+$-11 (2.26)

Stacking (S)

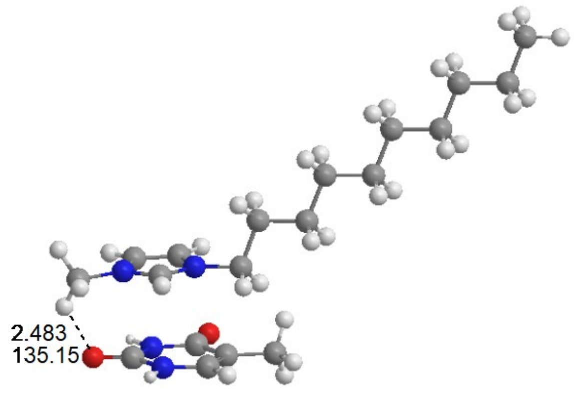

T-[C_{10}mim]$^{+}$-1 (0.00)

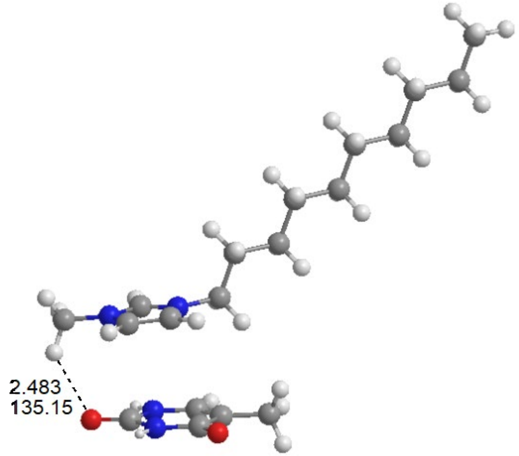

T-[C_{10}mim]$^{+}$-2 (0.34)

Perpendicular (P)

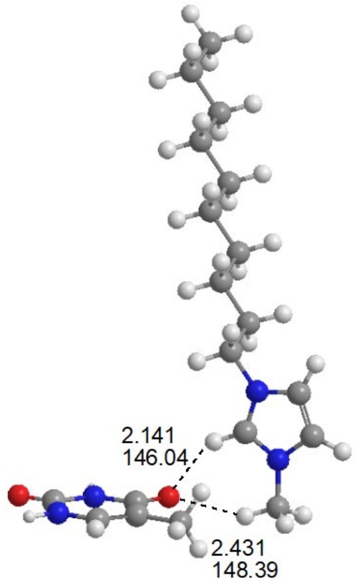

T-[C_{10}mim]$^{+}$-5 (0.00)

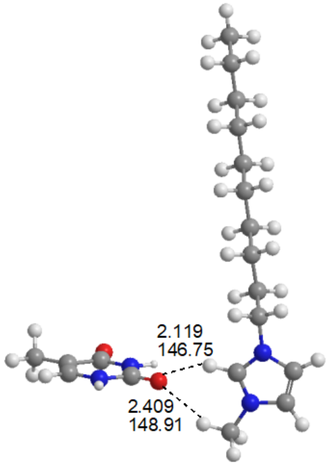

T-[C_{10}mim]$^{+}$-6 (1.45)

Coplanar (H)

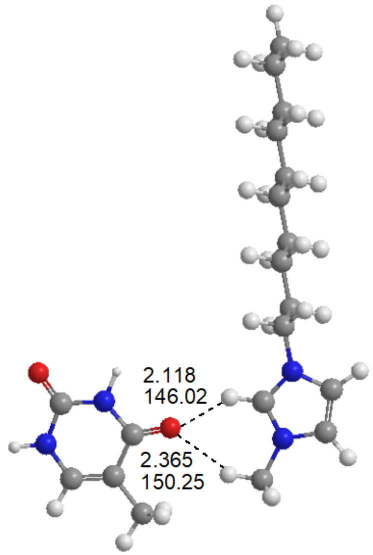

T-[C_{10}mim]$^{+}$-9 (0.00)

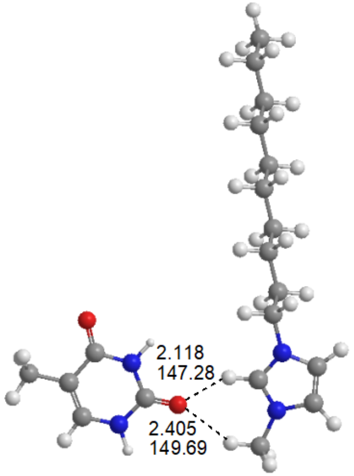

T-[C_{10}mim]$^{+}$-10 (1.18)

Continued

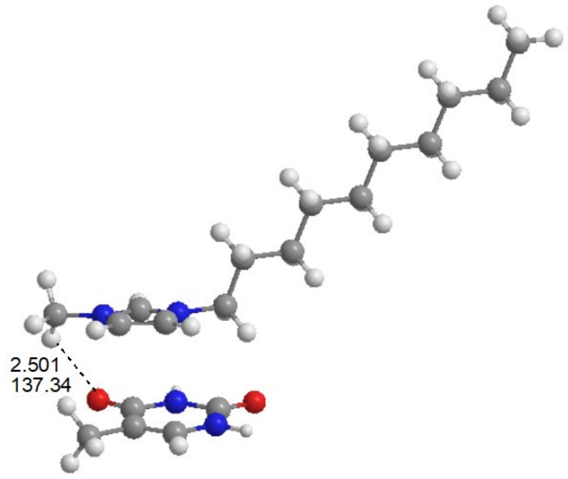

T-$[C_{10}mim]^+$-3 (1.15)

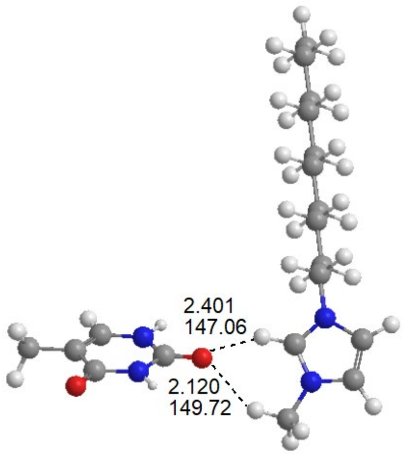

T-$[C_{10}mim]^+$-7 (2.91)

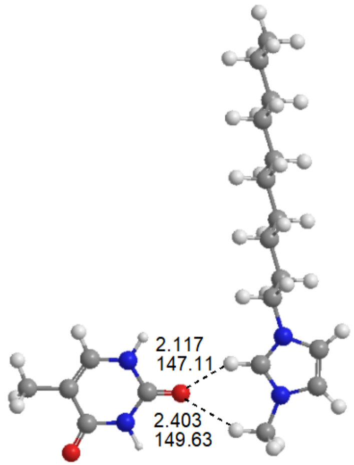

T-$[C_{10}mim]^+$-11 (1.49)

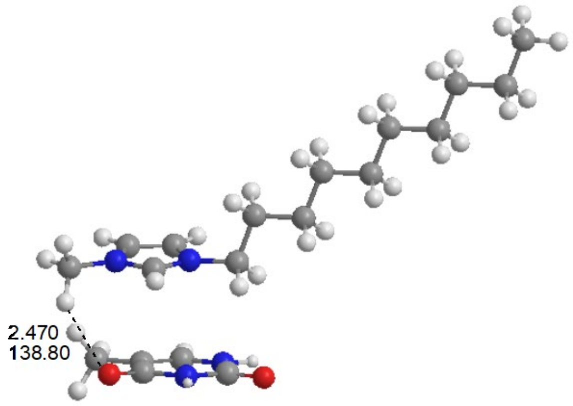

T-$[C_{10}mim]^+$-4 (1.42)

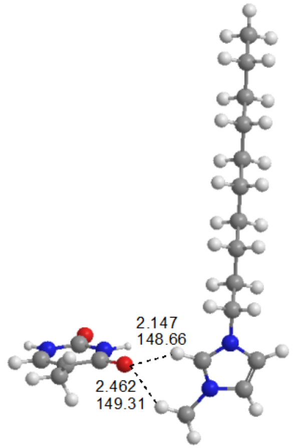

T-$[C_{10}mim]^+$-9 (3.45)

Figure S3. 11 of the mono-cationic structures of thymine with five imidazolium cations (T-$[C_nmim]^+$, n = 2, 4, 6, 8, 10) calculated at the M06-2X/6-311++G(2*d*, *p*)/PCM/water level of theory. The ZPVE-corrected stability decreases from top to bottom for each configuration (in kJ/mol).

Stacking (S) | **Perpendicular (P)** | **Coplanar (H)**

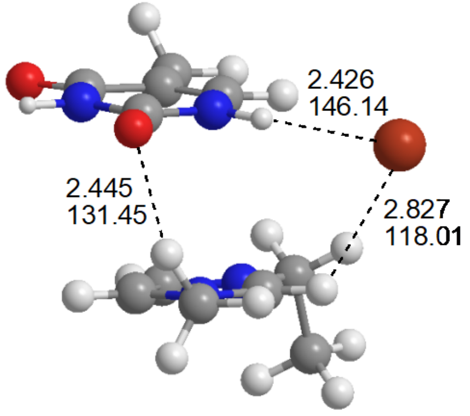

T-[C_2mim]$^+$-Br$^-$-1 (0.00)

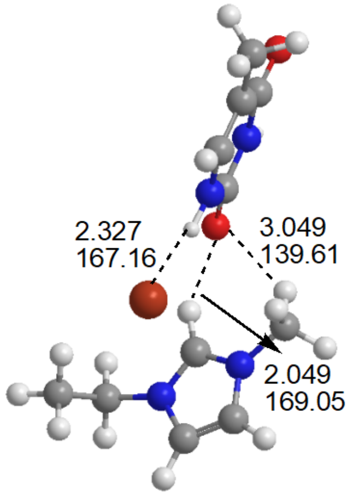

T-[C_2mim]$^+$-Br$^-$-5 (0.00)

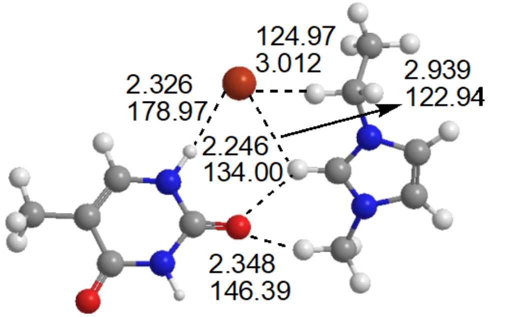

T-[C_2mim]$^+$-Br$^-$-9 (0.00)

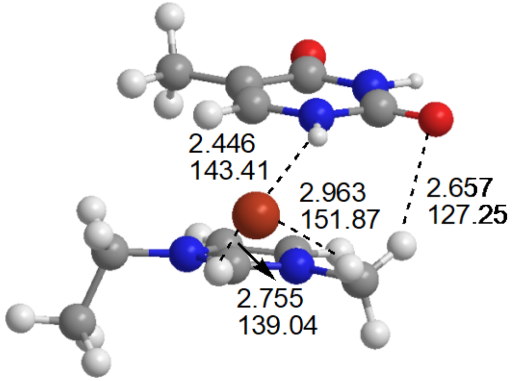

T-[C_2mim]$^+$-Br$^-$-2 (2.57)

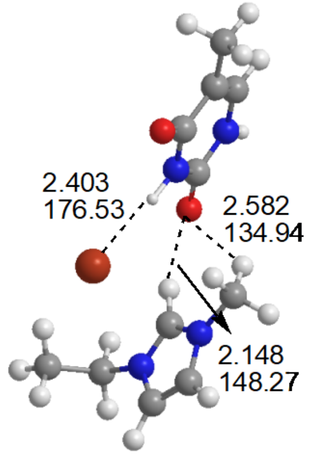

T-[C_2mim]$^+$-Br$^-$-6 (4.83)

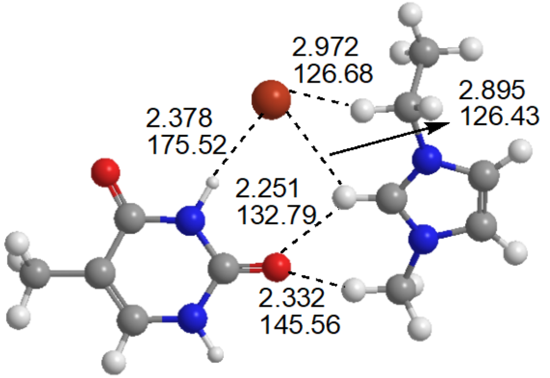

T-[C_2mim]$^+$-Br$^-$-10 (2.95)

Continued

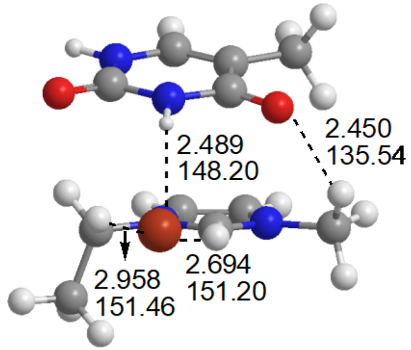

T-[C_2mim]$^+$-Br$^-$-3 (4.28)

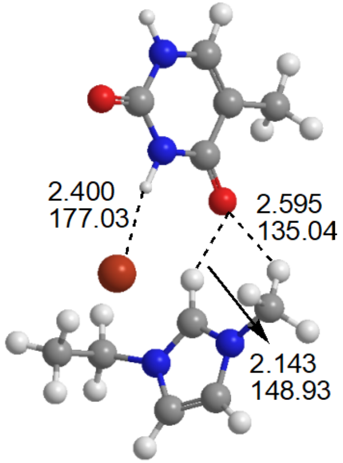

T-[C_2mim]$^+$-Br$^-$-7 (4.95)

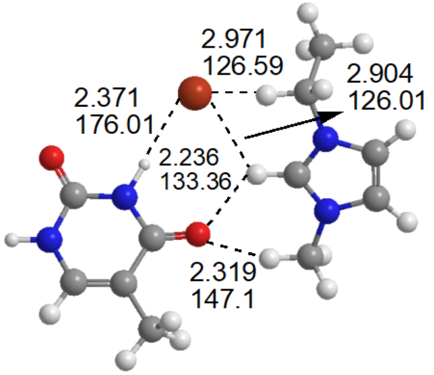

T-[C_2mim]$^+$-Br$^-$-11 (3.08)

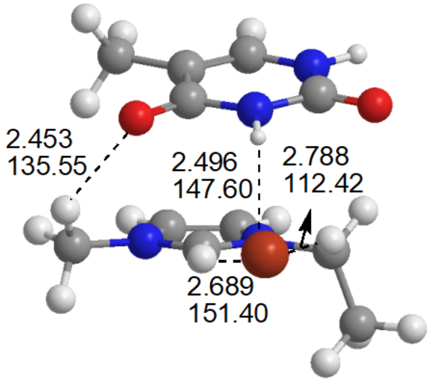

T-[C_2mim]$^+$-Br$^-$-4 (4.33)

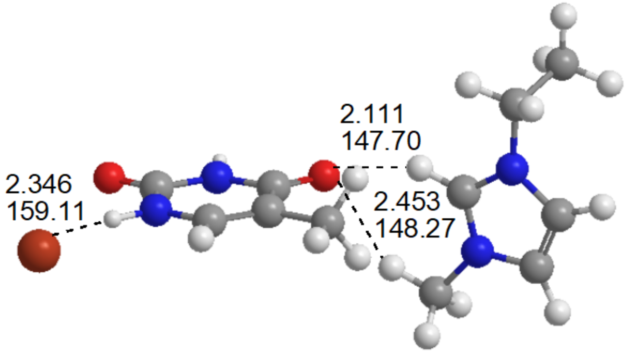

T-[C_2mim]$^+$-Br$^-$-8 (13.43)

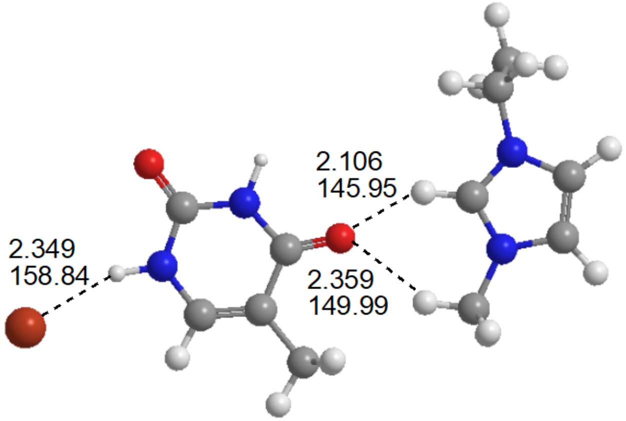

T-[C_2mim]$^+$-Br$^-$-12 (10.76)

Stacking (S)

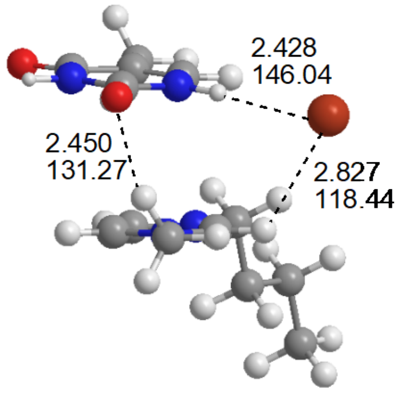

T-[C$_4$mim]$^+$-Br$^-$-1 (0.00)

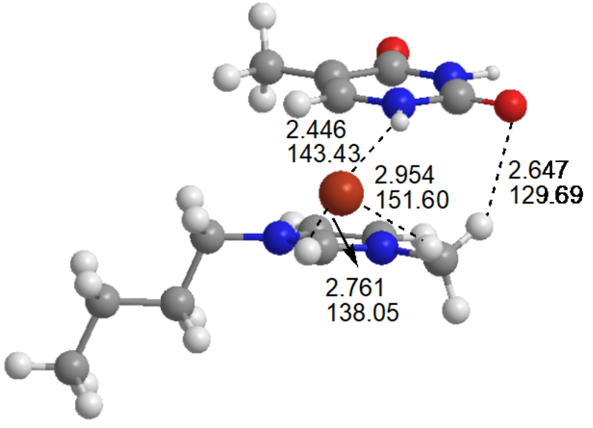

T-[C$_4$mim]$^+$-Br$^-$-2 (2.27)

Perpendicular (P)

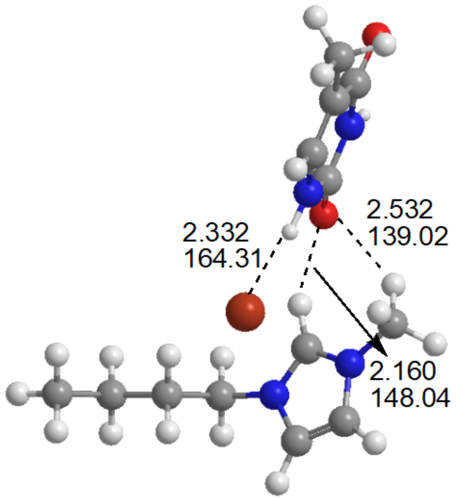

T-[C$_4$mim]$^+$-Br$^-$-5 (0.00)

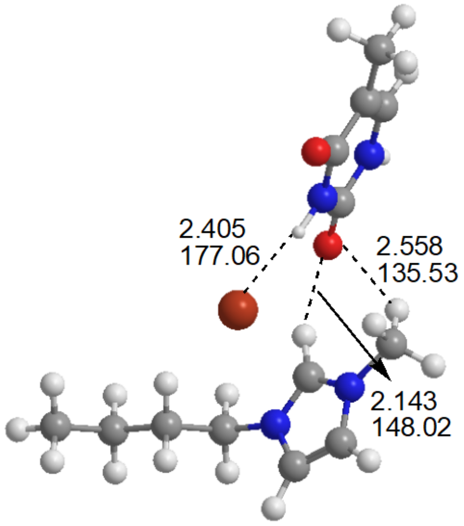

T-[C$_4$mim]$^+$-Br$^-$-6 (4.21)

Coplanar (H)

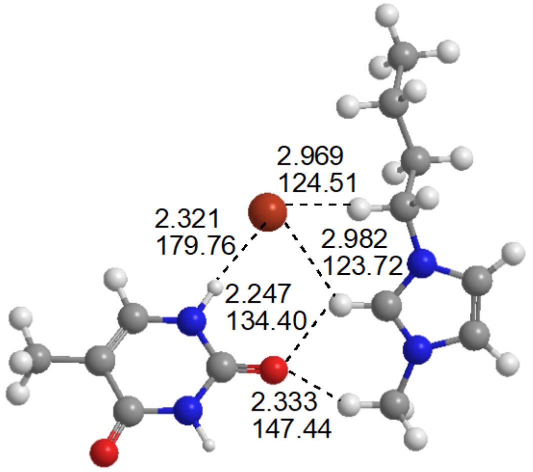

T-[C$_4$mim]$^+$-Br$^-$-9 (0.00)

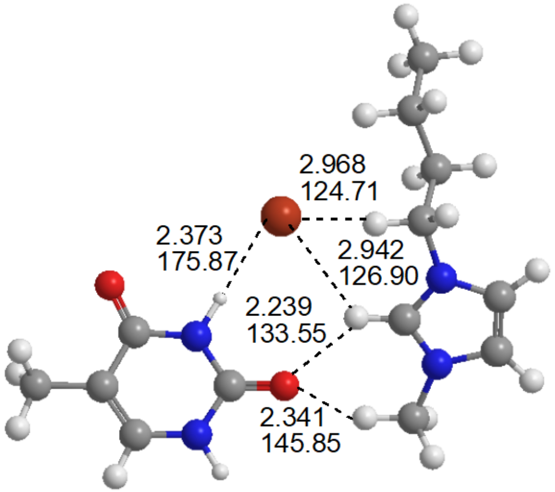

T-[C$_4$mim]$^+$-Br$^-$-10 (3.23)

Continued

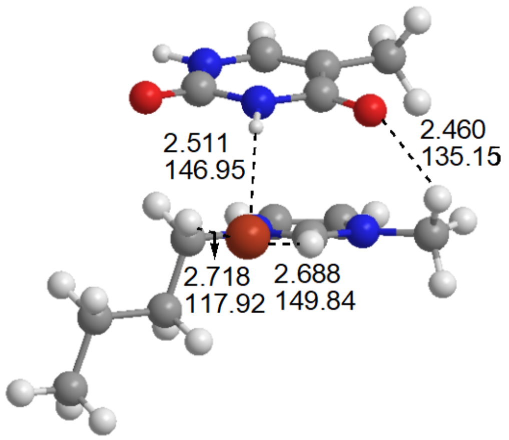

T-[C4mim]+-Br−-3 (3.92)

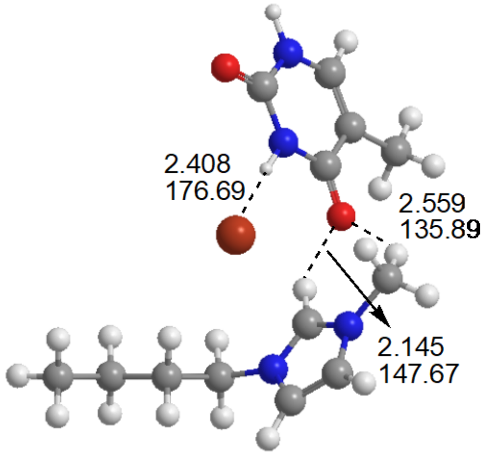

T-[C4mim]+-Br−-7 (5.00)

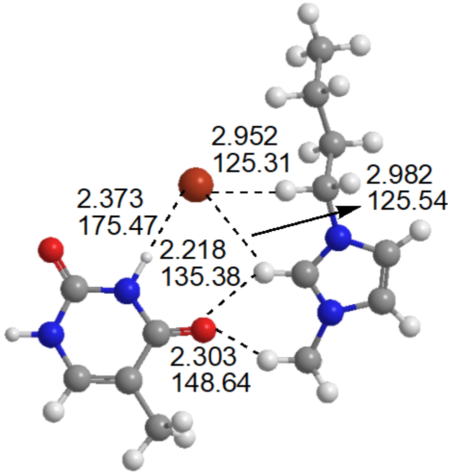

T-[C4mim]+-Br−-11 (3.66)

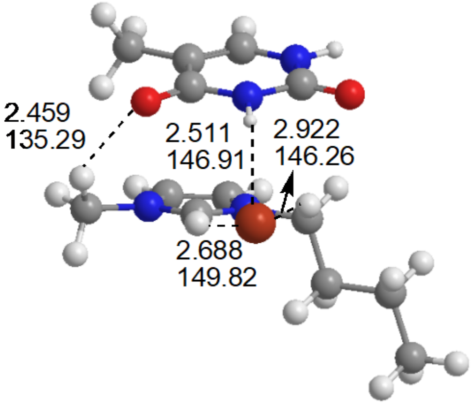

T-[C4mim]+-Br−-4 (3.92)

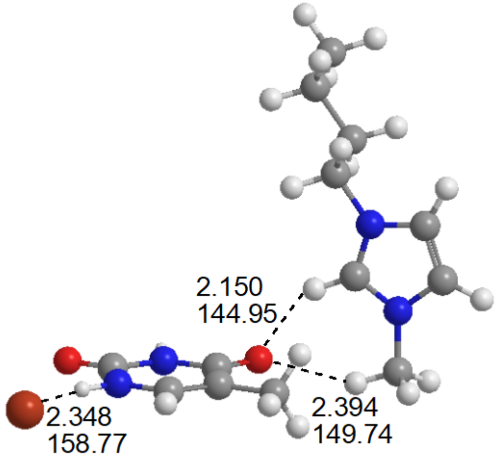

T-[C4mim]+-Br−-8 (14.25)

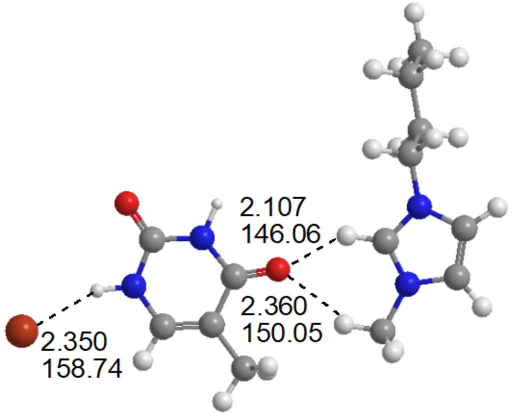

T-[C4mim]+-Br−-12 (12.77)

Stacking (S) | **Perpendicular (P)** | **Coplanar (H)**

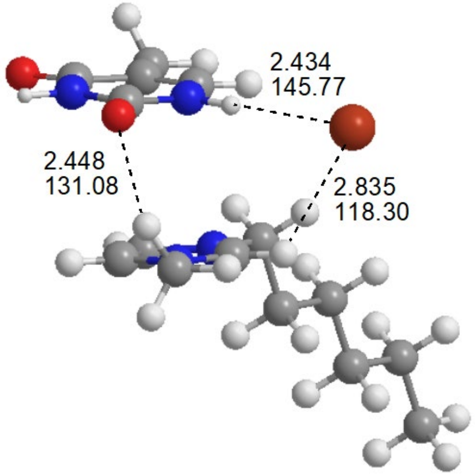

T-[C_6mim]$^+$-Br$^-$-1 (0.00)

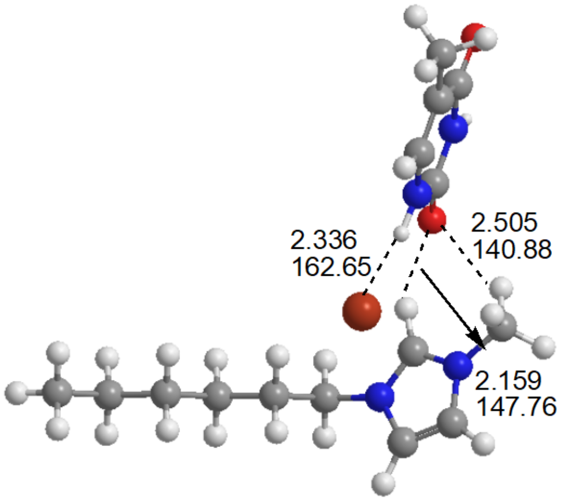

T-[C_6mim]$^+$-Br$^-$-5 (0.00)

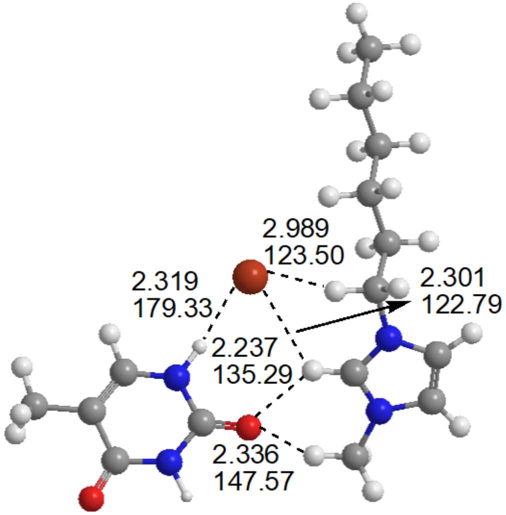

T-[C_6mim]$^+$-Br$^-$-9 (0.00)

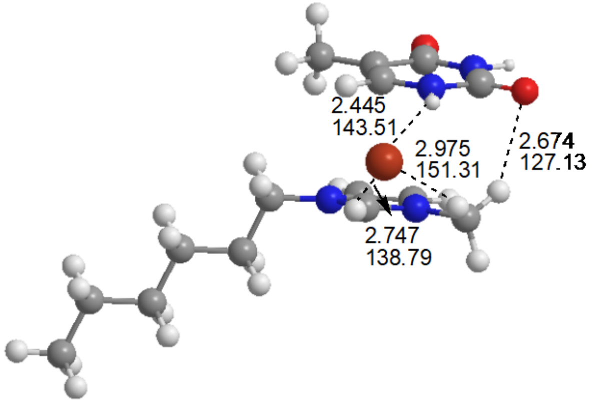

T-[C_6mim]$^+$-Br$^-$-2 (2.42)

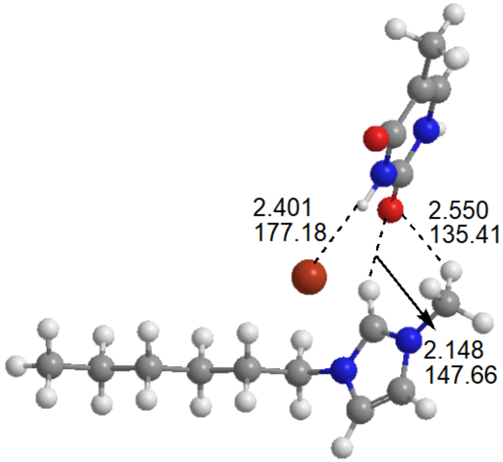

T-[C_6mim]$^+$-Br$^-$-6 (4.36)

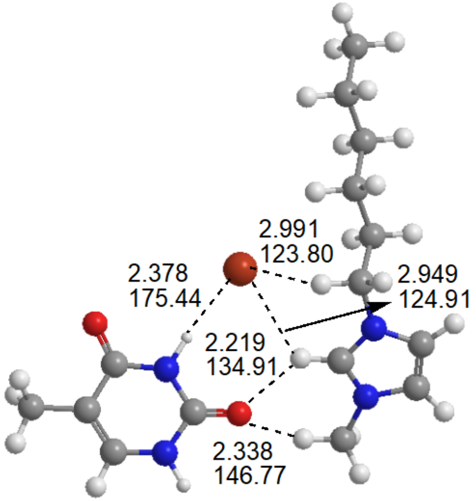

T-[C_6mim]$^+$-Br$^-$-10 (2.81)

Continued

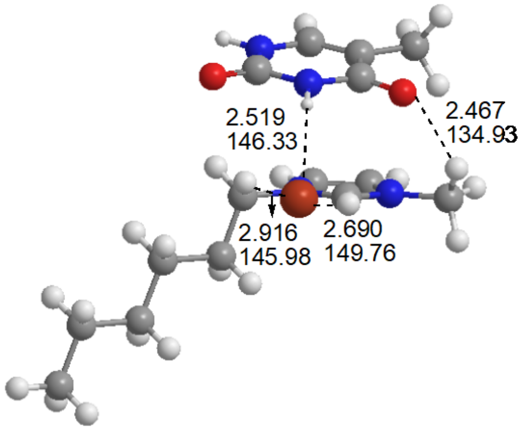

T-[C$_6$mim]$^+$-Br$^-$-3 (4.07)

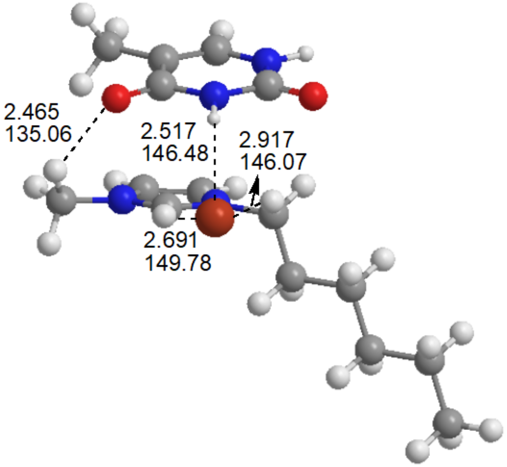

T-[C$_6$mim]$^+$-Br$^-$-4 (4.08)

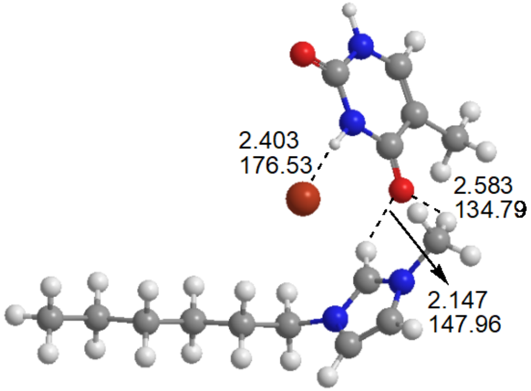

T-[C$_6$mim]$^+$-Br$^-$-7 (4.86)

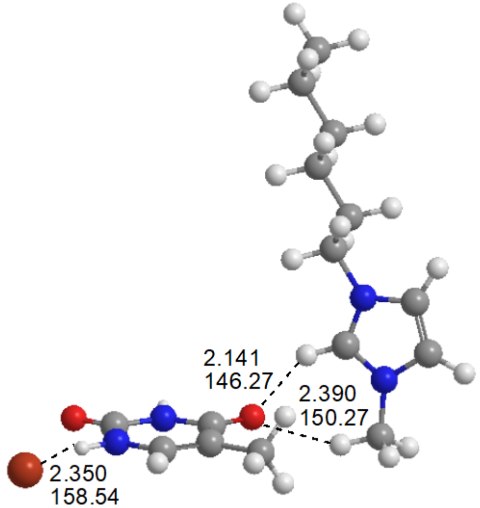

T-[C$_6$mim]$^+$-Br$^-$-8 (14.20)

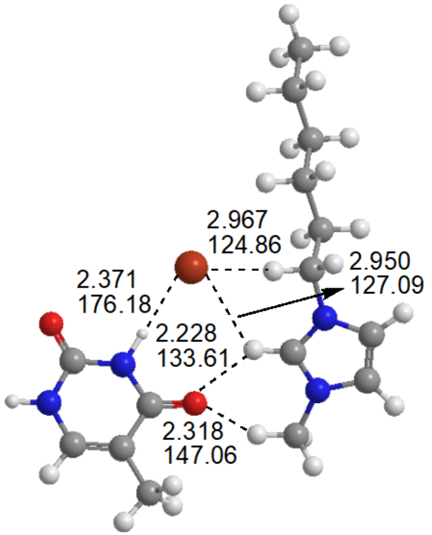

T-[C$_6$mim]$^+$-Br$^-$-11 (3.52)

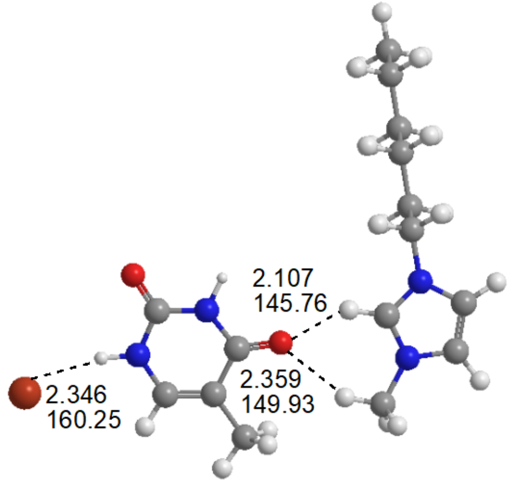

T-[C$_6$mim]$^+$-Br$^-$-12 (12.58)

Stacking (S)

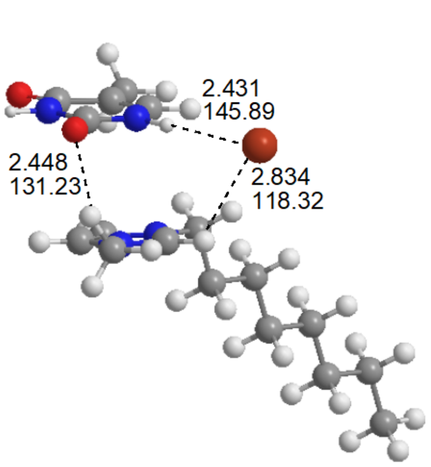

T-[C8mim]$^{+}$-Br^{-}-1 (0.00)

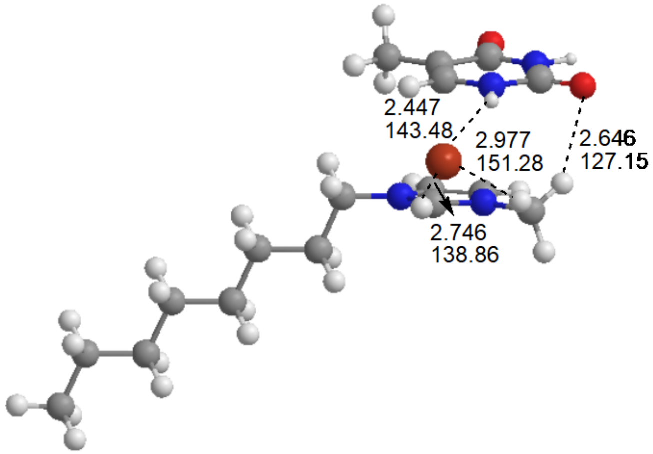

T-[C8mim]$^{+}$-Br^{-}-2 (2.33)

Perpendicular (P)

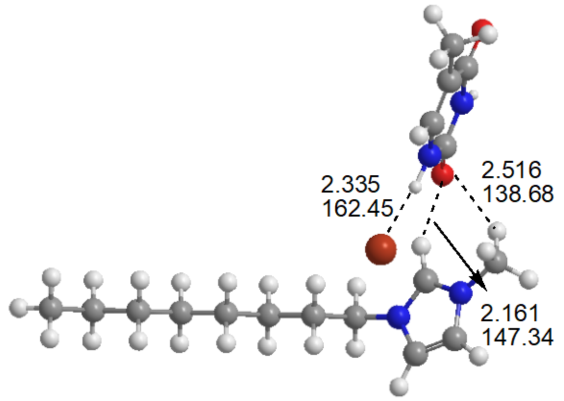

T-[C8mim]$^{+}$-Br^{-}-5 (0.00)

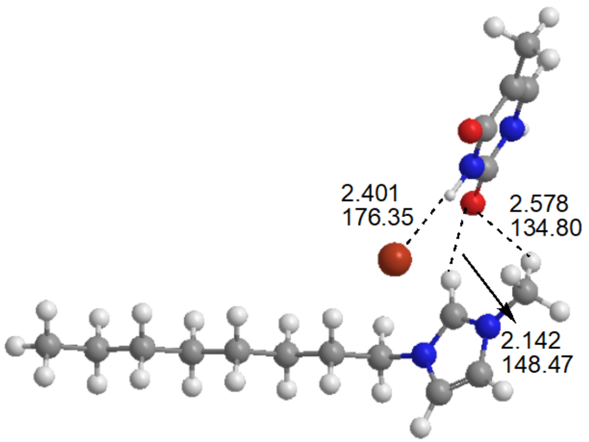

T-[C8mim]$^{+}$-Br^{-}-6 (4.20)

Coplanar (H)

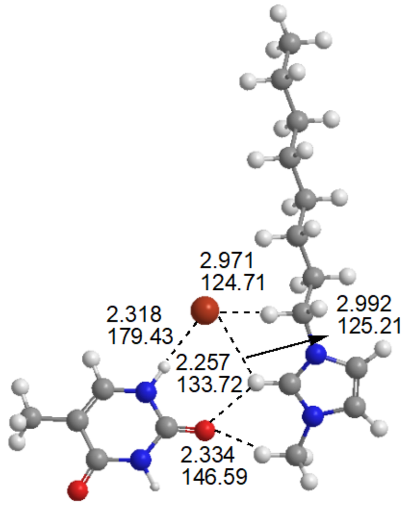

T-[C8mim]$^{+}$-Br^{-}-9 (0.00)

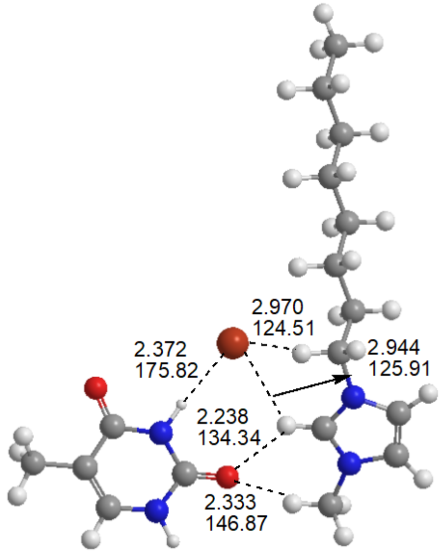

T-[C8mim]$^{+}$-Br^{-}-10 (3.81)

Continued

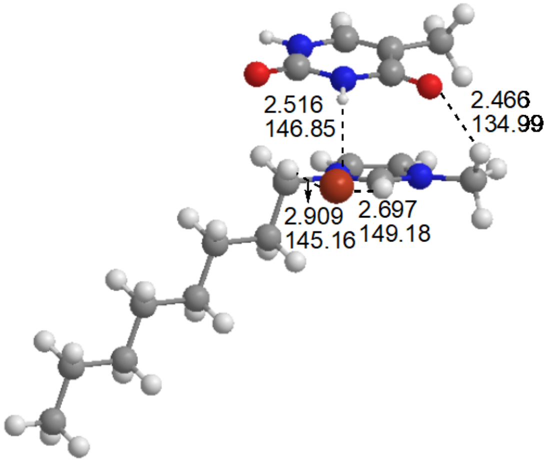

T-[C$_8$mim]$^+$-Br$^-$-3 (4.20)

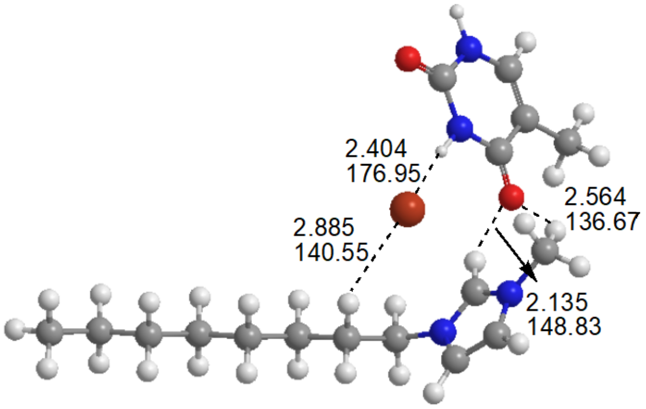

T-[C$_8$mim]$^+$-Br$^-$-7 (5.04)

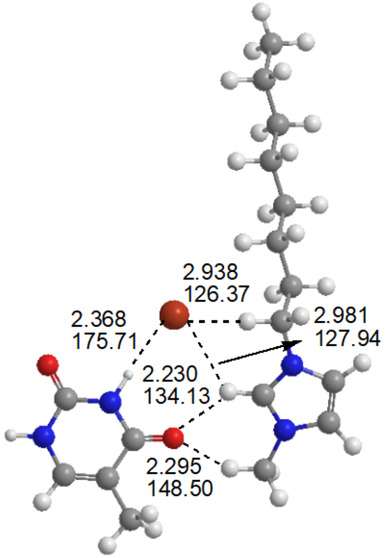

T-[C$_8$mim]$^+$-Br$^-$-11 (4.10)

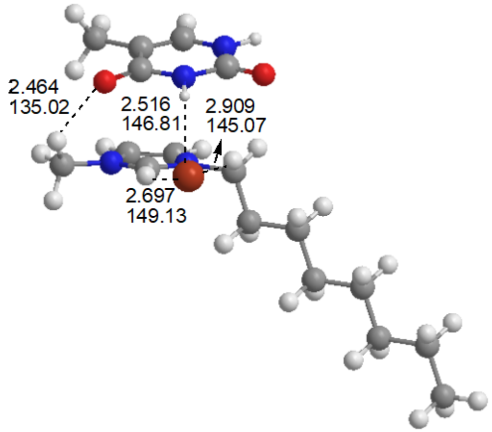

T-[C$_8$mim]$^+$-Br$^-$-4 (4.26)

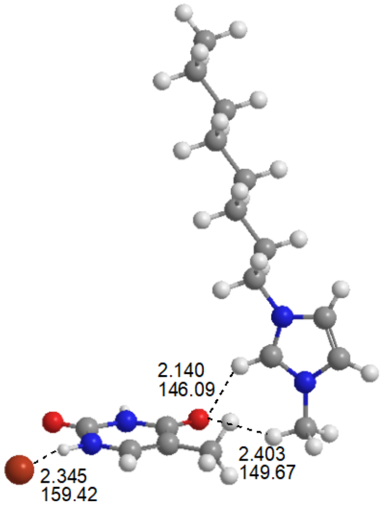

T-[C$_8$mim]$^+$-Br$^-$-8 (14.02)

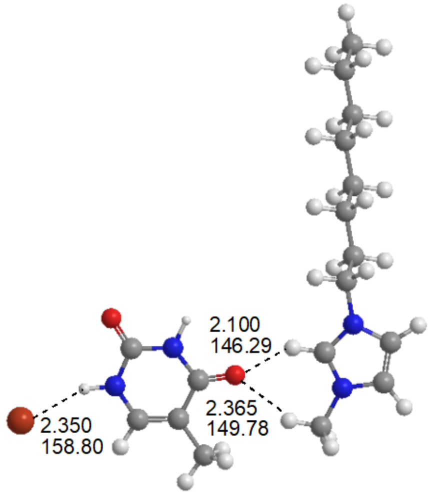

T-[C$_8$mim]$^+$-Br$^-$-12 (13.76)

Stacking (S)

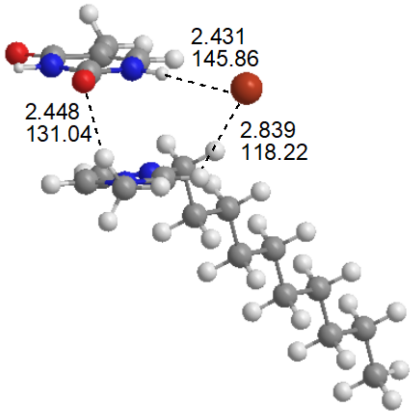

T-[C_{10}mim]$^+$-Br$^-$-1 (0.00)

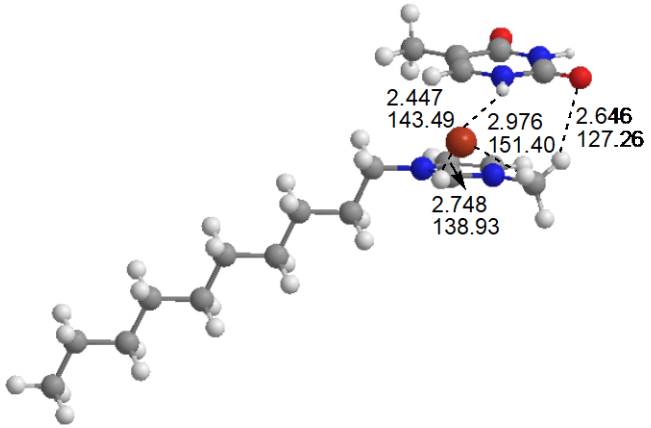

T-[C_{10}mim]$^+$-Br$^-$-2 (3.07)

Perpendicular (P)

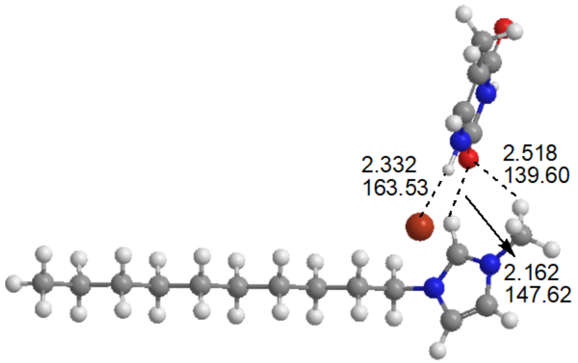

T-[C_{10}mim]$^+$-Br$^-$-5 (0.00)

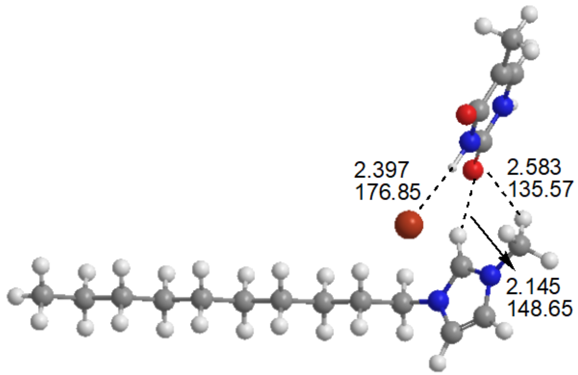

T-[C_{10}mim]$^+$-Br$^-$-6 (4.31)

Coplanar (H)

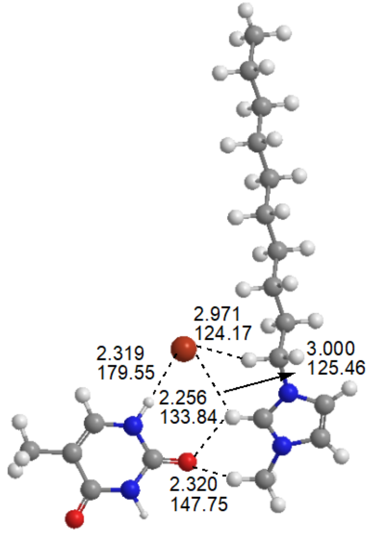

T-[C_{10}mim]$^+$-Br$^-$-9 (0.00)

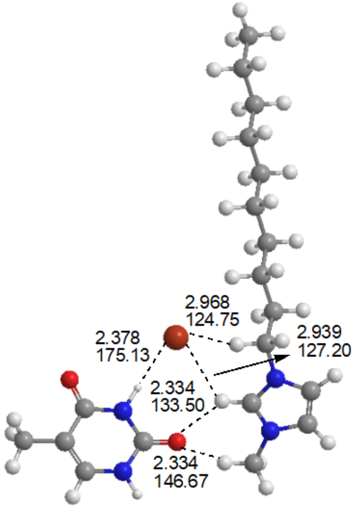

T-[C_{10}mim]$^+$-Br$^-$-10 (3.15)

Continued

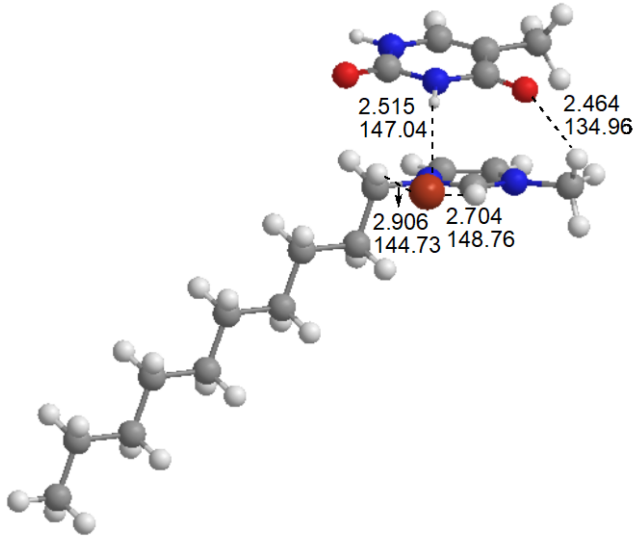

T-$[C_{10}mim]^+$-Br^--3 (4.09)

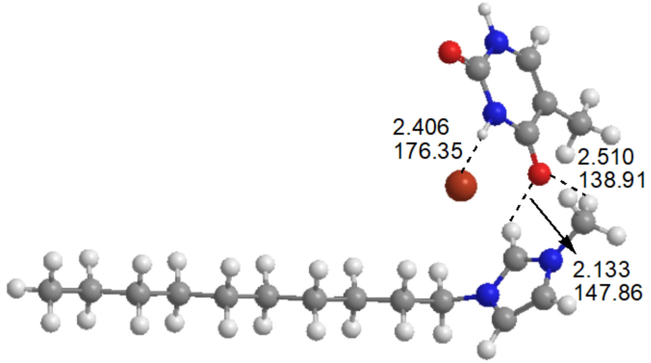

T-$[C_{10}mim]^+$-Br^--7 (4.91)

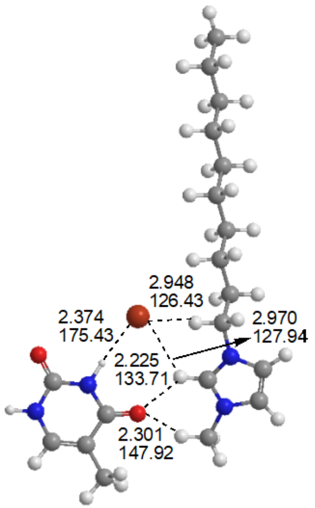

T-$[C_{10}mim]^+$-Br^--11 (3.67)

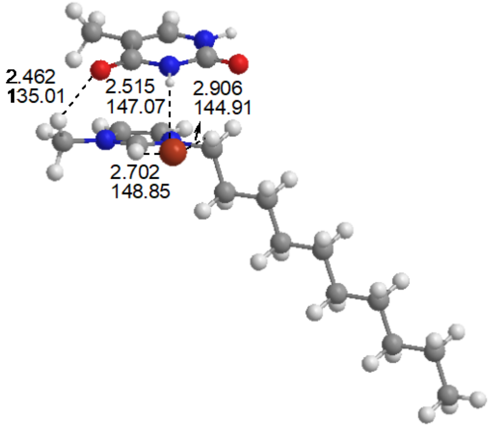

T-$[C_{10}mim]^+$-Br^--4 (4.27)

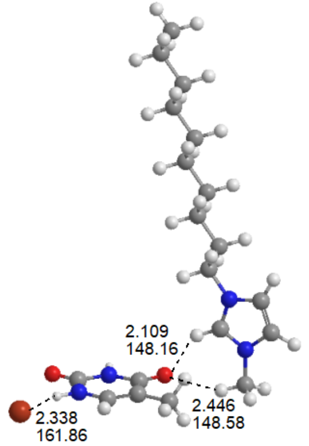

T-$[C_{10}mim]^+$-Br^--8 (12.80)

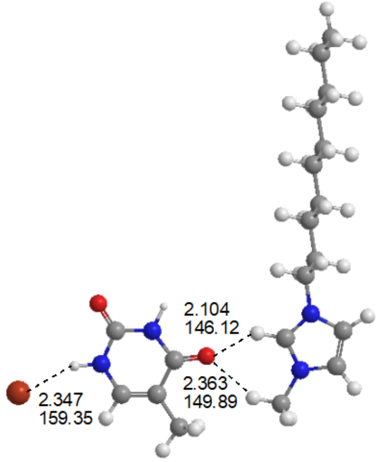

T-$[C_{10}mim]^+$-Br^--12 (13.12)

Figure S4. 12 of the di-ionic structures of thymine with five imidazolium ILs (T-$[C_nmim]^+$-Br^- , n = 2, 4, 6, 8, 10) calculated at the M06-2X/6-311++G(2*d*, *p*)/PCM/water level of theory. The ZPVE-corrected stability decreases from top to bottom for each configuration (in kJ/mol).

Stacking (S)	Perpendicular (P)	Coplanar (H)

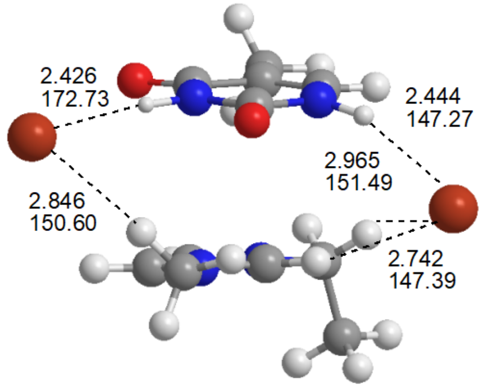

T-$[C_2mim]^+$-2Br^--1 (0.00)

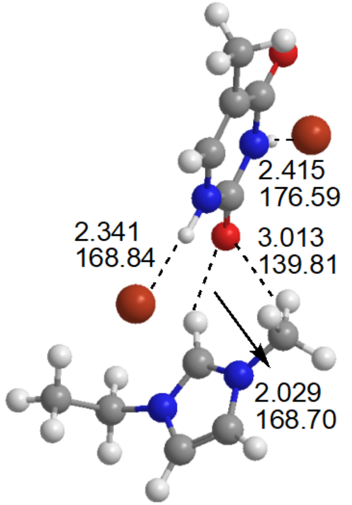

T-$[C_2mim]^+$-2Br^--2 (15.75)

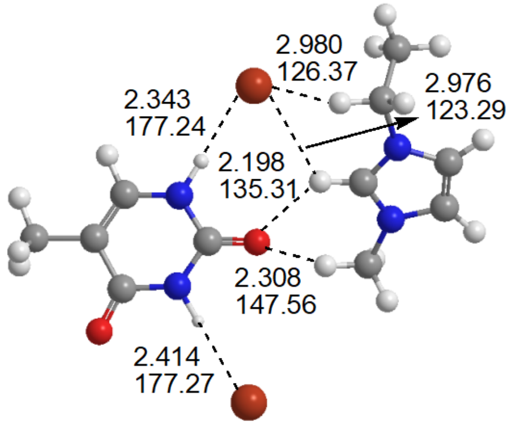

T-$[C_2mim]^+$-2Br^--3 (18.95)

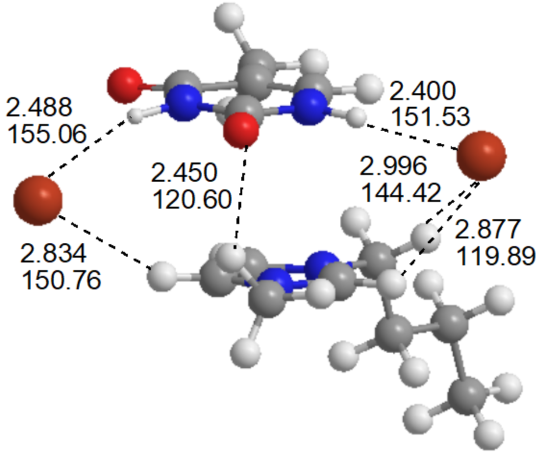

T-$[C_4mim]^+$-2Br^--1 (0.00)

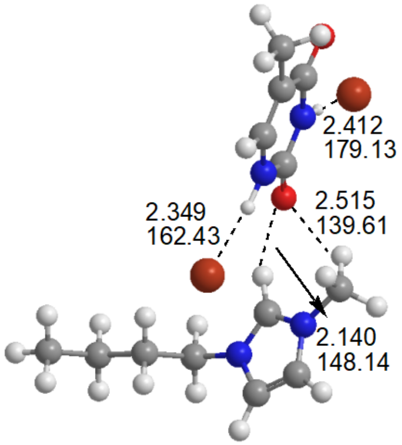

T-$[C_4mim]^+$-2Br^--2 (11.77)

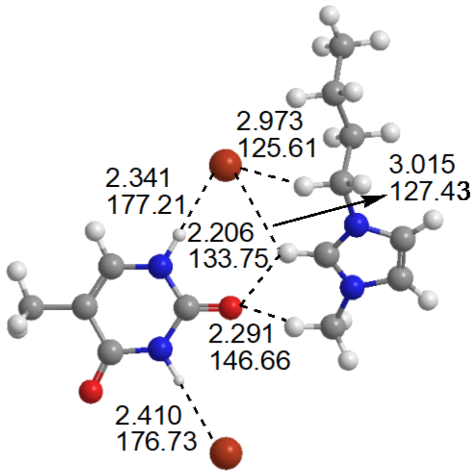

T-$[C_4mim]^+$-2Br^--3 (13.14)

Continued

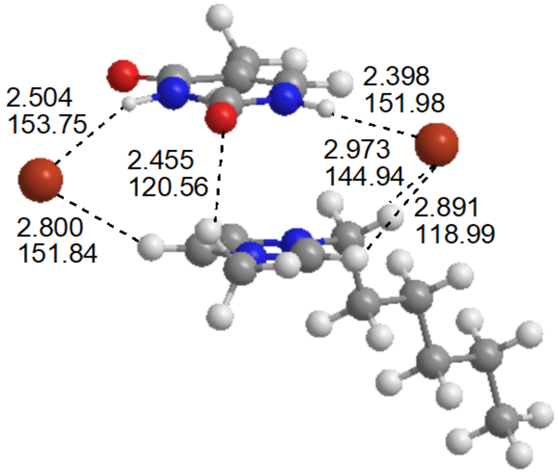

T-[C$_6$mim]$^+$-2Br$^-$-1 (0.00)

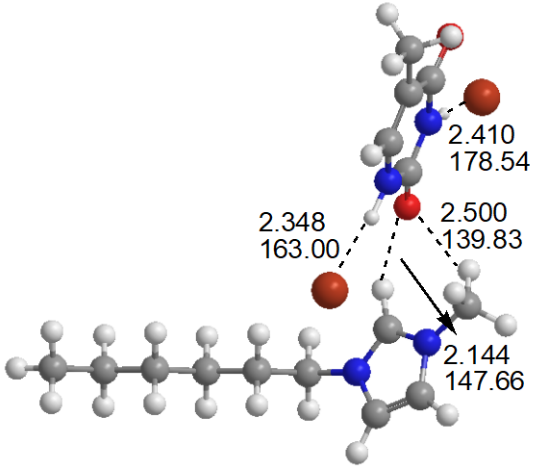

T-[C$_6$mim]$^+$-2Br$^-$-2 (10.76)

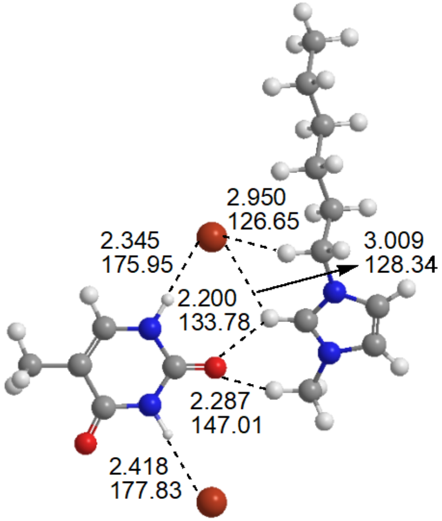

T-[C$_6$mim]$^+$-2Br$^-$-3 (12.05)

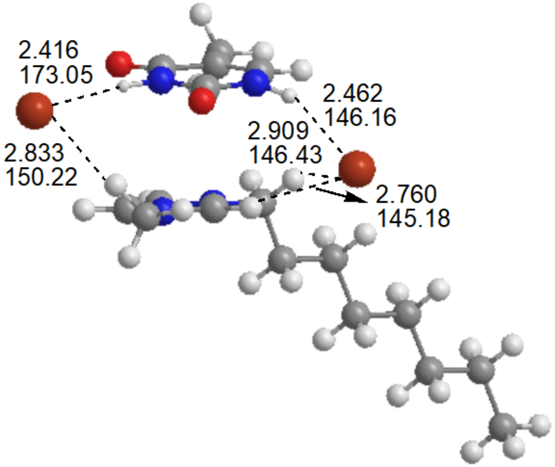

T-[C$_8$mim]$^+$-2Br$^-$-1 (0.00)

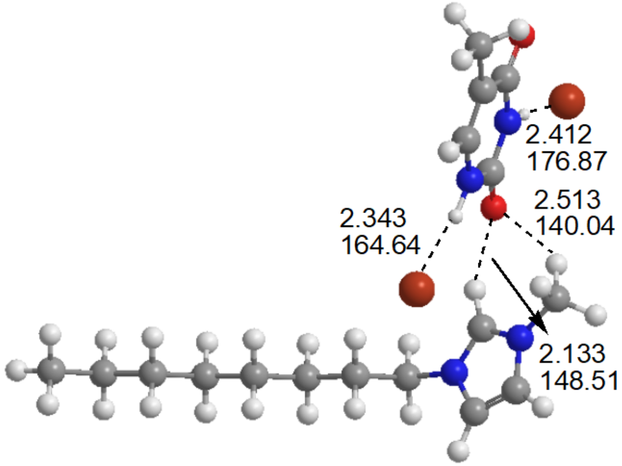

T-[C$_8$mim]$^+$-2Br$^-$-2 (17.92)

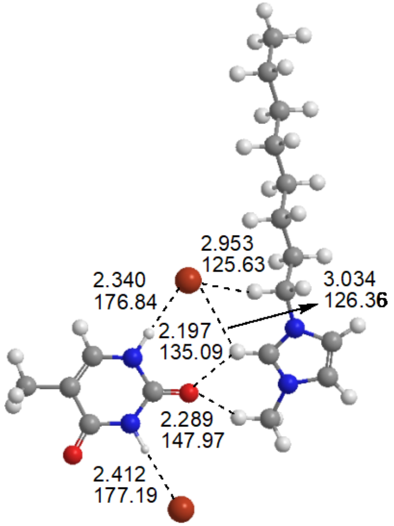

T-[C$_8$mim]$^+$-2Br$^-$-3 (18.55)

Continued

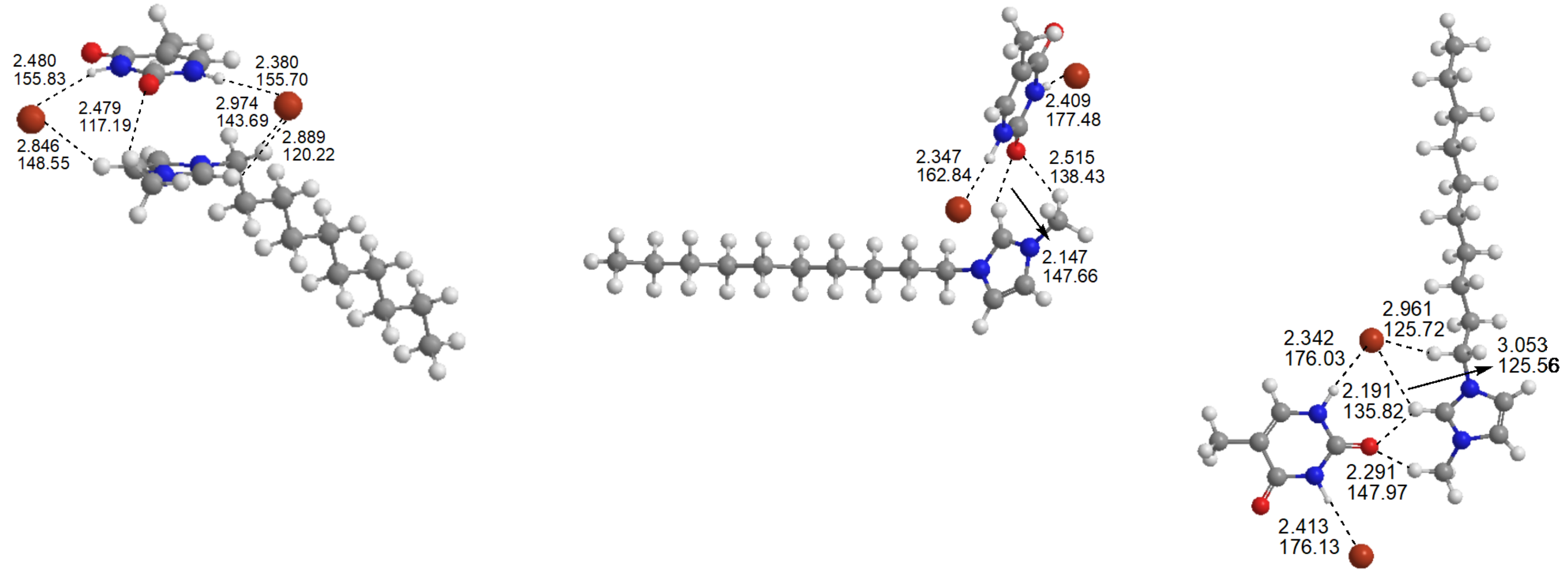

T-[C_{10}mim]$^+$-2Br$^-$-1 (0.00) T-[C_{10}mim]$^+$-2Br$^-$-2 (12.53) T-[C_{10}mim]$^+$-2Br$^-$-3 (12.62)

Figure S5. 3 of the tri-ionic structures of thymine with five imidazolium ILs (T-[$C_n$mim]$^+$-2Br$^-$, n = 2, 4, 6, 8, 10) based on the stacking, perpendicular, and coplanar configurations and calculated at the M06-2X/6-311++G(2*d*, *p*)/PCM/water level of theory. The ZPVE-corrected stability decreases from left to right (in kJ/mol).

Stacking (S) | **Perpendicular (P)** | **Coplanar (H)**

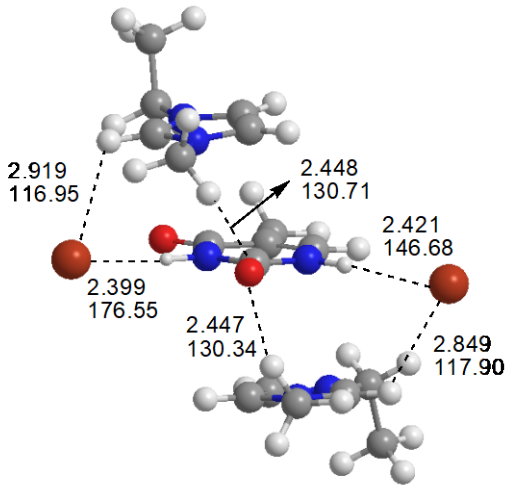

T-2[C_2mim]$^+$-2Br$^-$-1 (0.00)

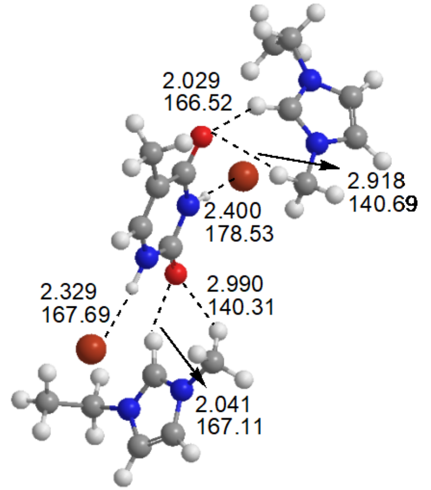

T-2[C_2mim]$^+$-2Br$^-$-2 (18.69)

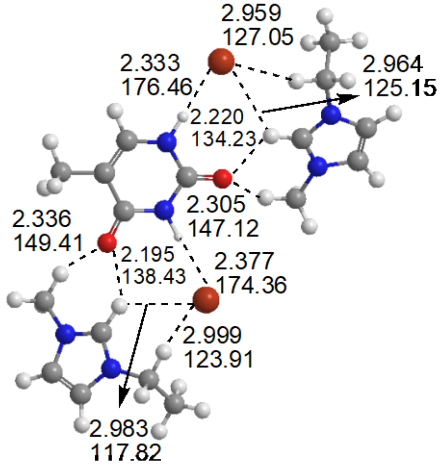

T-2[C_2mim]$^+$-2Br$^-$-3 (25.38)

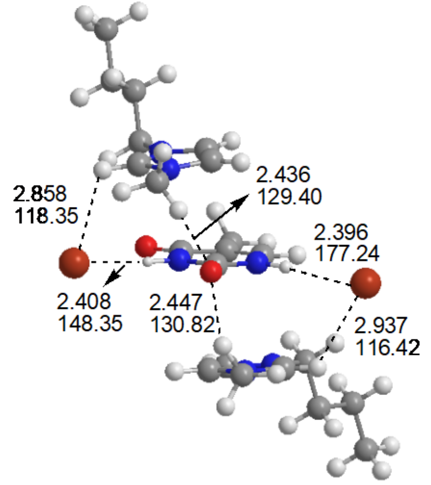

T-2[C_4mim]$^+$-2Br$^-$-1 (0.00)

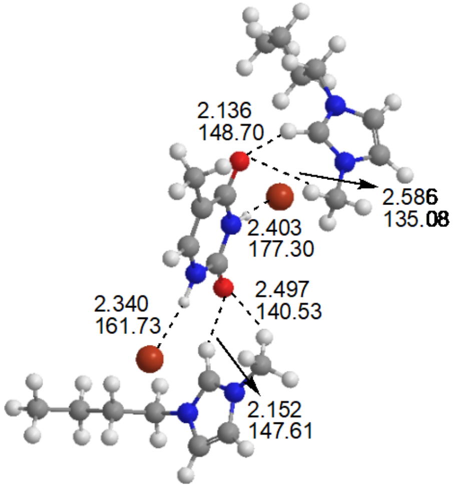

T-2[C_4mim]$^+$-2Br$^-$-2 (19.17)

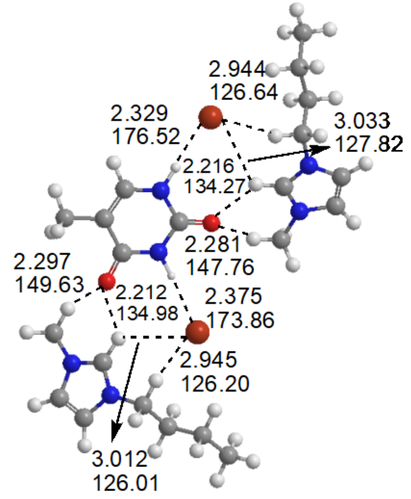

T-2[C_4mim]$^+$-2Br$^-$-3 (21.95)

Continued

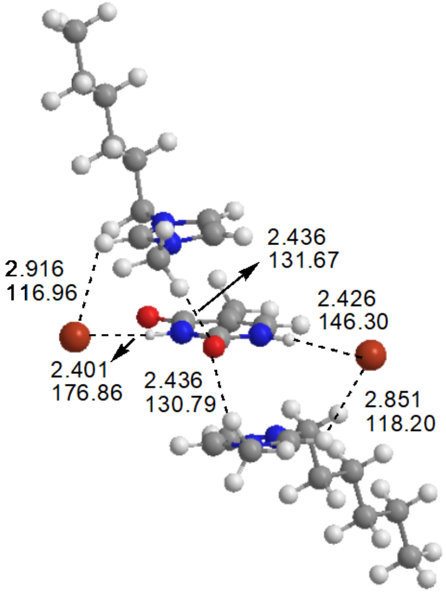

T-2[C_6mim]$^+$-2Br$^-$-1 (0.00)

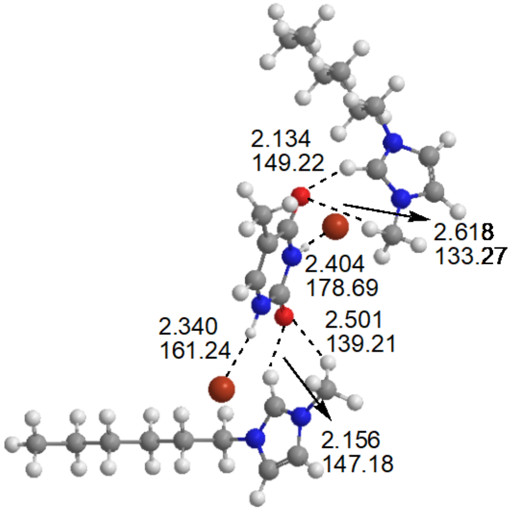

T-2[C_6mim]$^+$-2Br$^-$-2 (19.13)

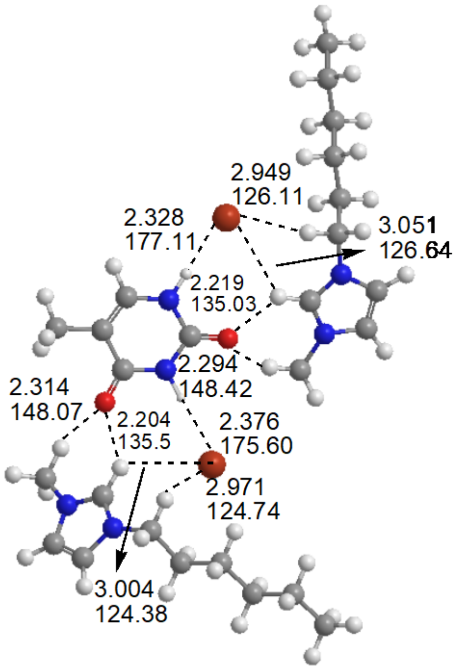

T-2[C_6mim]$^+$-2Br$^-$-3 (21.04)

Continued

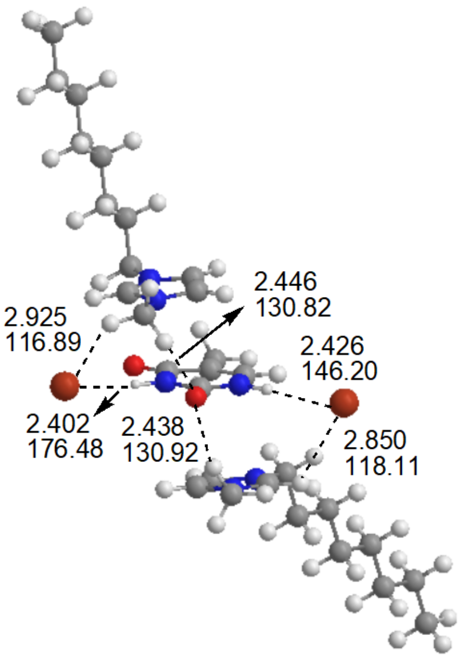

T-2[C_8mim]$^+$-2Br$^-$-1 (0.00)

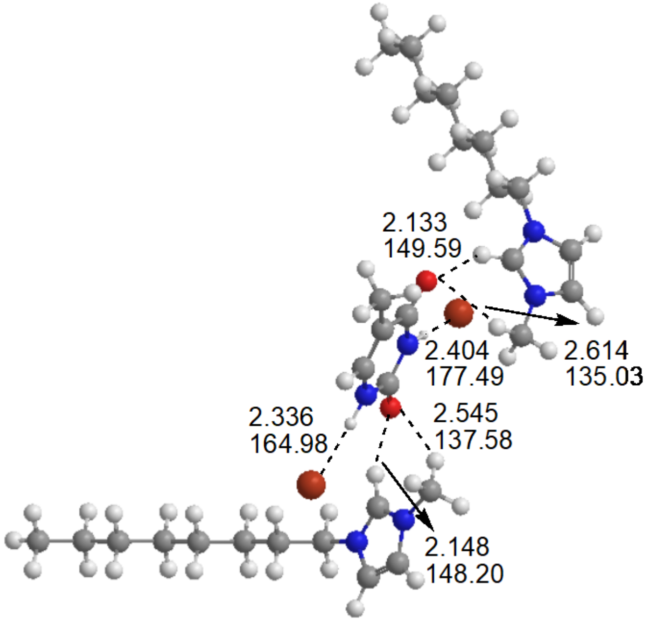

T-2[C_8mim]$^+$-2Br$^-$-2 (20.20)

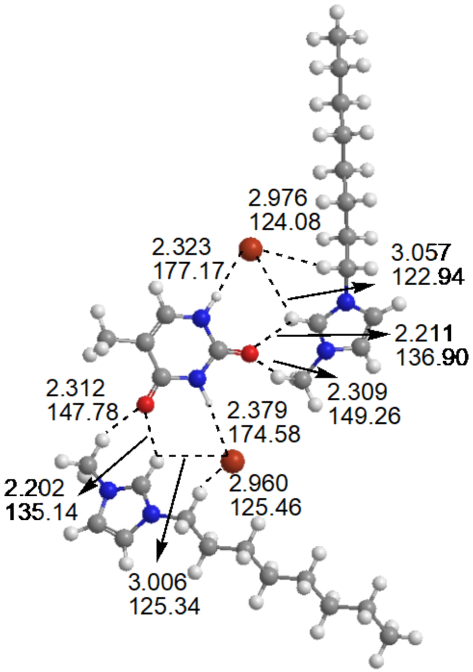

T-2[C_8mim]$^+$-2Br$^-$-3 (21.60)

Continued

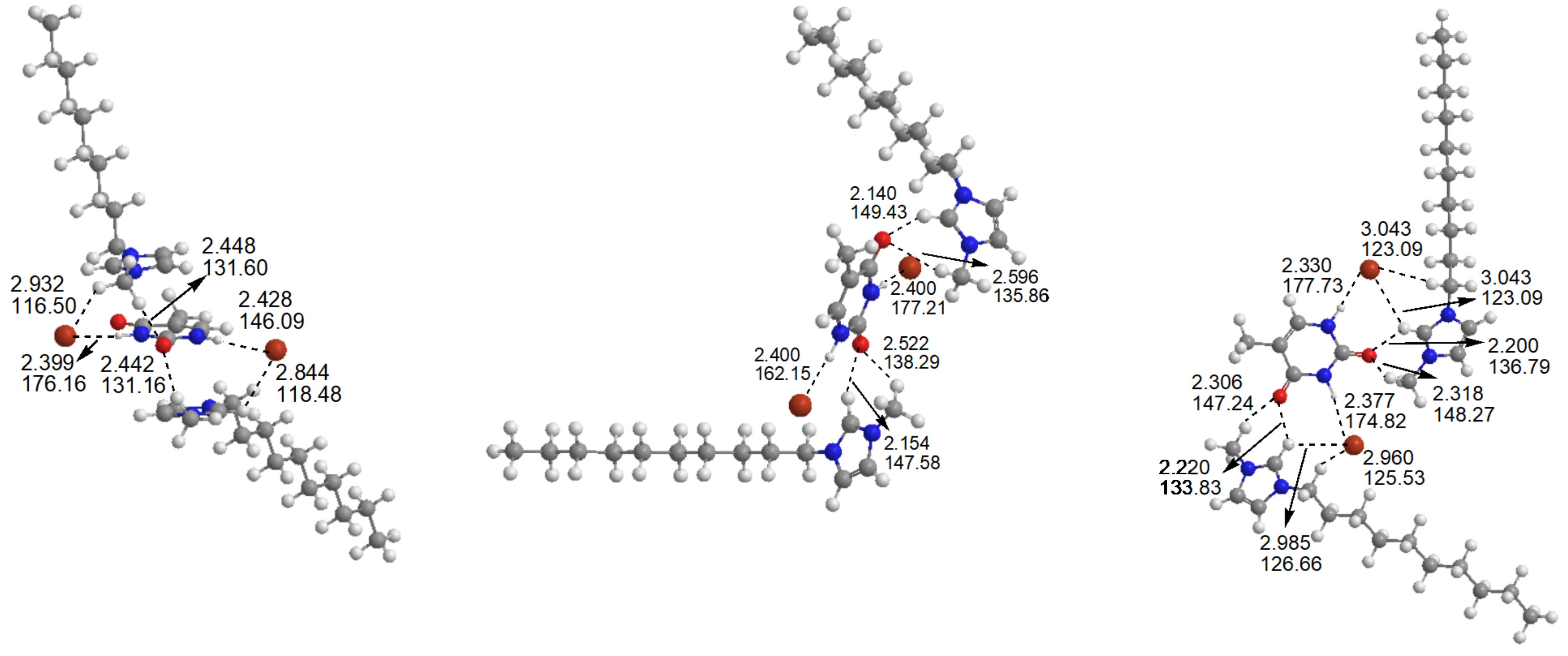

T-2[C$_{10}$mim]$^+$-2Br$^-$-1 (0.00) T-2[C$_{10}$mim]$^+$-2Br$^-$-2 (19.91) T-2[C$_{10}$mim]$^+$-2Br$^-$-3 (21.57)

Figure S6. 3 of the tetra-ionic structures of thymine with five imidazolium ILs (T-2[C$_n$mim]$^+$-2Br$^-$, n = 2, 4, 6, 8, 10) based on the stacking, perpendicular, and coplanar configurations and calculated at the M06-2X/6-311++G(2*d*, *p*)/PCM/water level of theory. The ZPVE-corrected stability decreases from left to right (in kJ/mol).

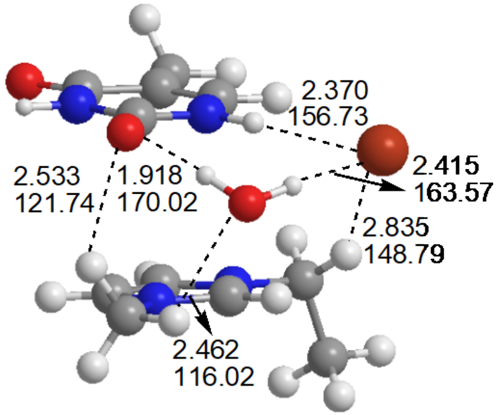

T-1H_2O-[C_2mim]$^+$-Br^--1 (0.00)

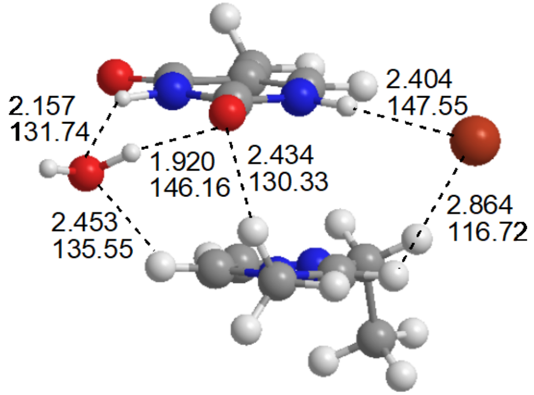

T-1H_2O-[C_2mim]$^+$-Br^--2 (5.59)

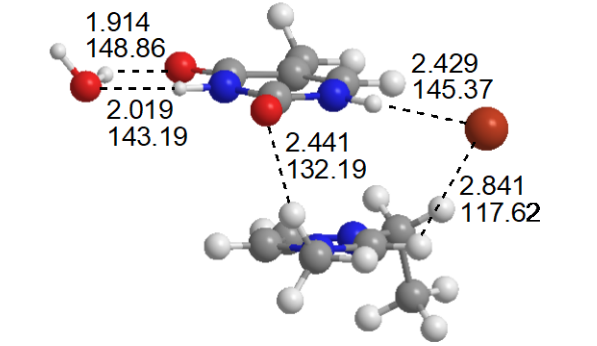

T-1H_2O-[C_2mim]$^+$-Br^--3 (7.05)

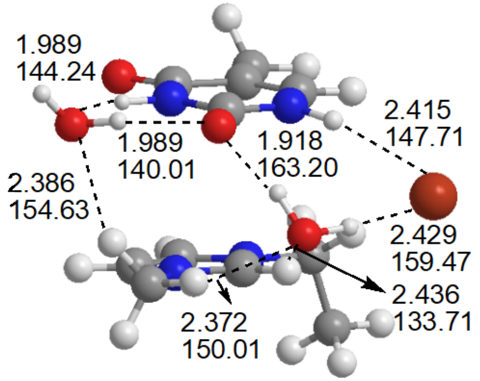

T-2H_2O-[C_2mim]$^+$-Br^--1 (0.00)

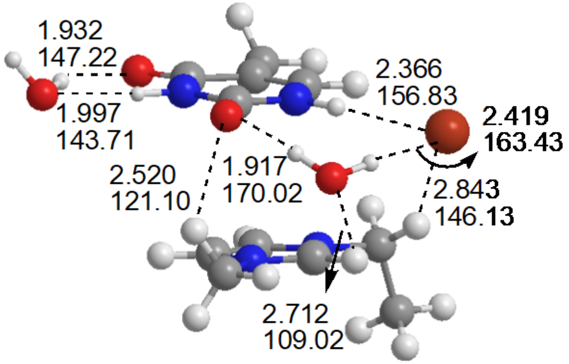

T-2H_2O-[C_2mim]$^+$-Br^--2 (1.67)

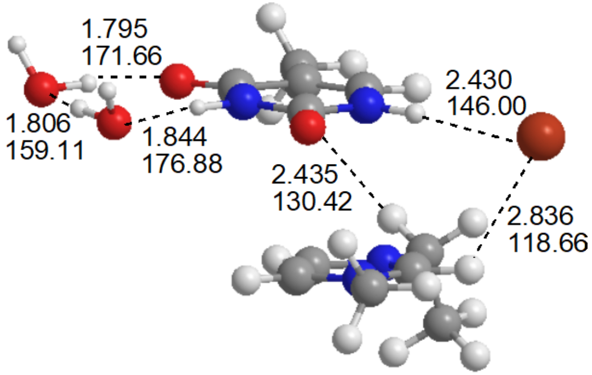

T-2H_2O-[C_2mim]$^+$-Br^--3 (3.85)

Continued

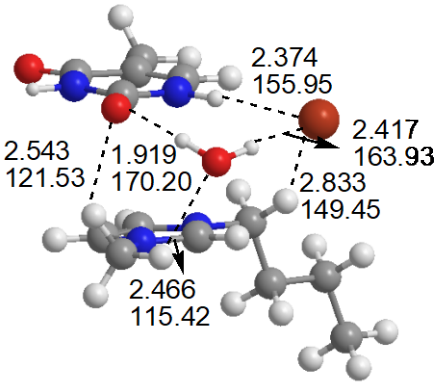

T-1H_2O-[C_4mim]$^+$-Br^--1 (0.00)

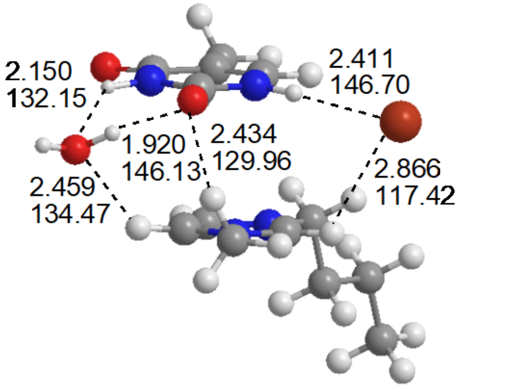

T-1H_2O-[C_4mim]$^+$-Br^--2 (6.87)

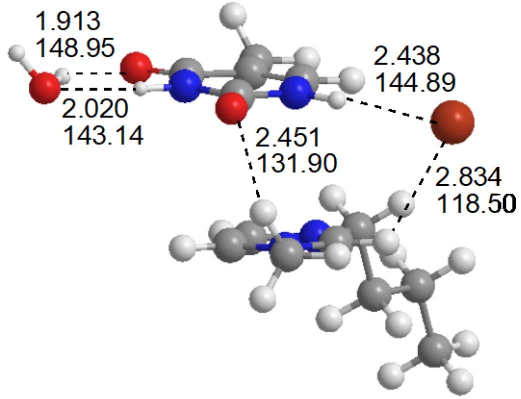

T-1H_2O-[C_4mim]$^+$-Br^--3 (7.91)

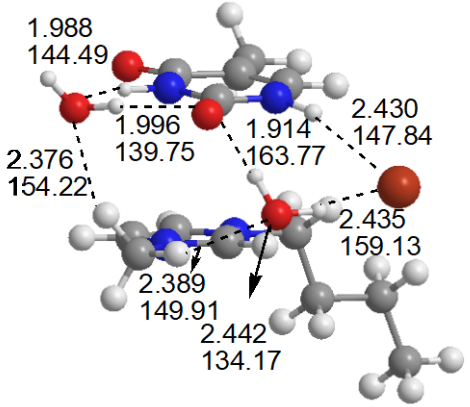

T-2H_2O-[C_4mim]$^+$-Br^--1 (0.00)

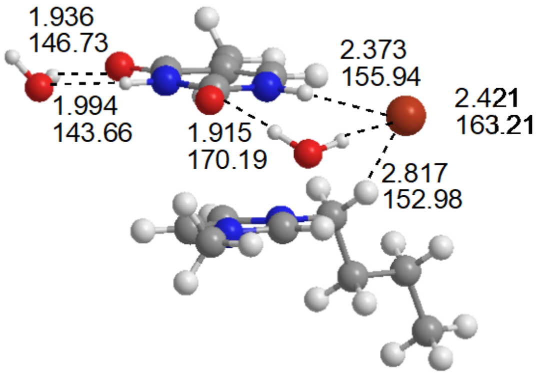

T-2H_2O-[C_4mim]$^+$-Br^--2 (2.21)

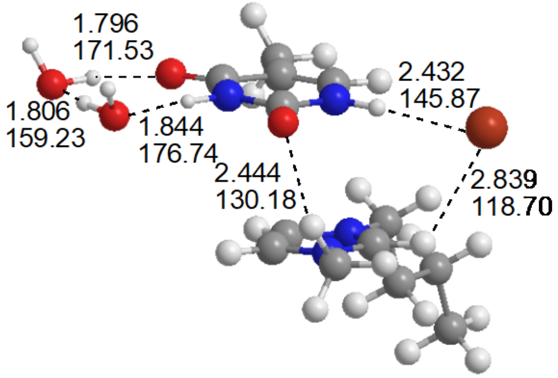

T-2H_2O-[C_4mim]$^+$-Br^--3 (4.88)

Continued

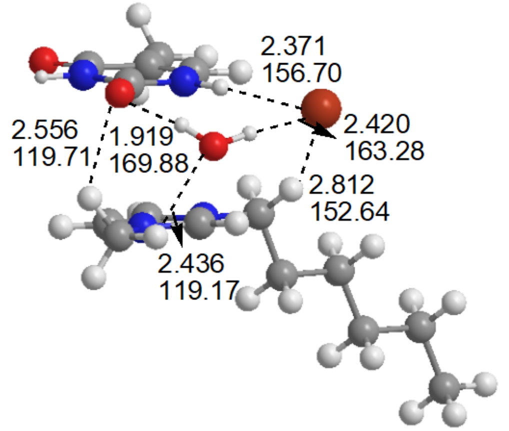

T-1H_2O-[C_6mim]$^+$-Br^--1 (0.00)

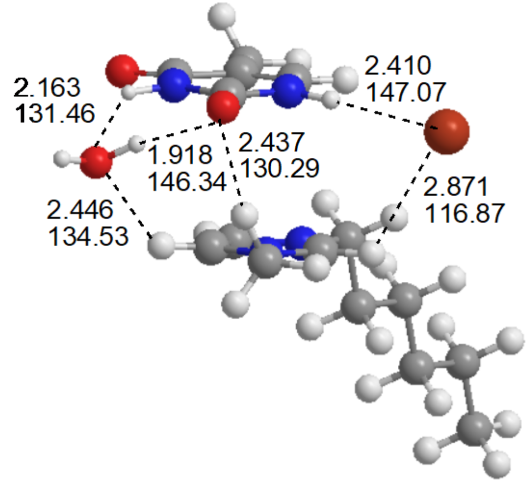

T-1H_2O-[C_6mim]$^+$-Br^--2 (7.16)

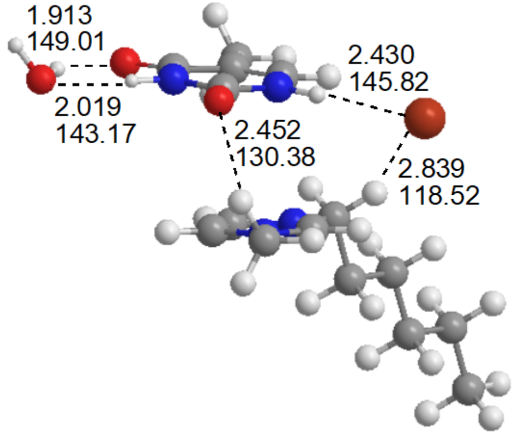

T-1H_2O-[C_6mim]$^+$-Br^--3 (8.66)

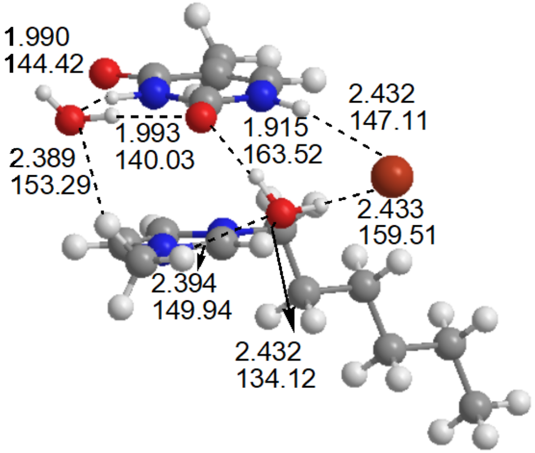

T-2H_2O-[C_6mim]$^+$-Br^--1 (0.00)

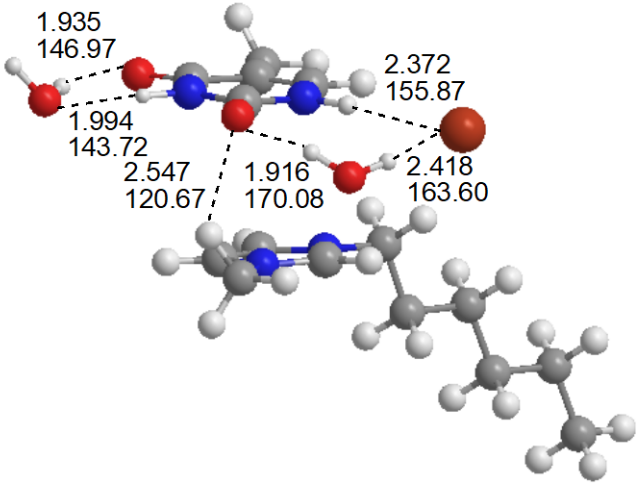

T-2H_2O-[C_6mim]$^+$-Br^--2 (2.25)

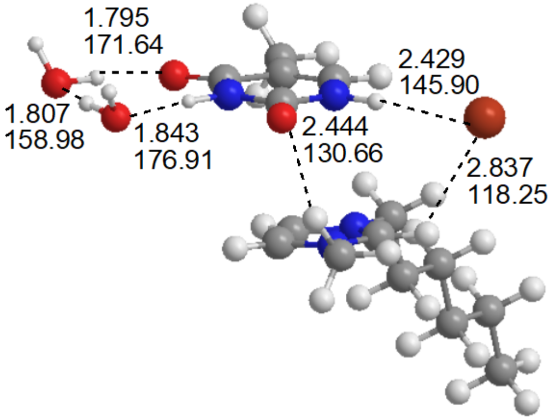

T-2H_2O-[C_6mim]$^+$-Br^--3 (5.77)

Continued

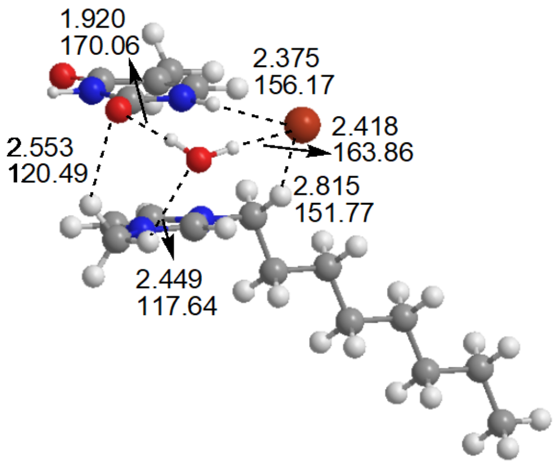

T-1H_2O-[C_8mim]$^+$-Br^--1 (0.00)

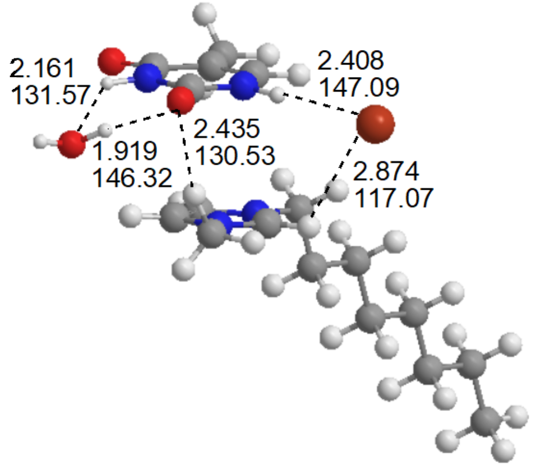

T-1H_2O-[C_8mim]$^+$-Br^--2 (6.89)

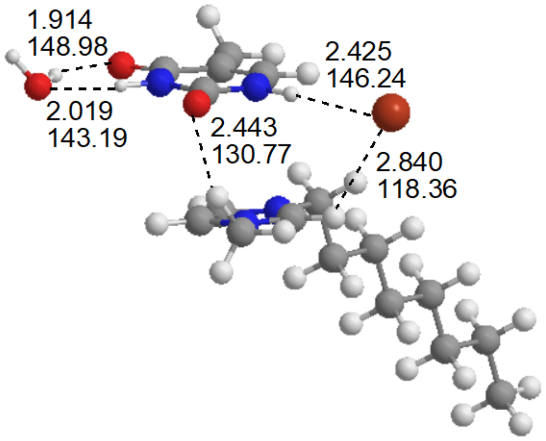

T-1H_2O-[C_8mim]$^+$-Br^--3 (8.29)

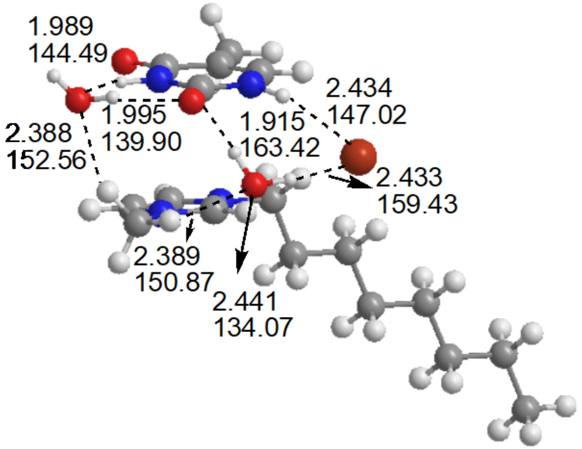

T-2H_2O-[C_8mim]$^+$-Br^--1 (0.00)

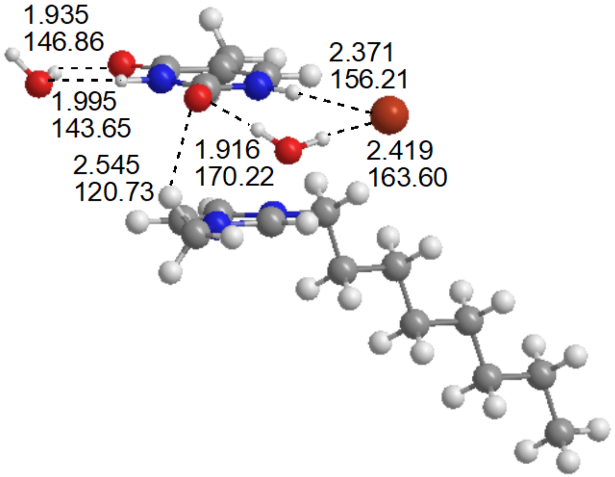

T-2H_2O-[C_8mim]$^+$-Br^--2 (3.30)

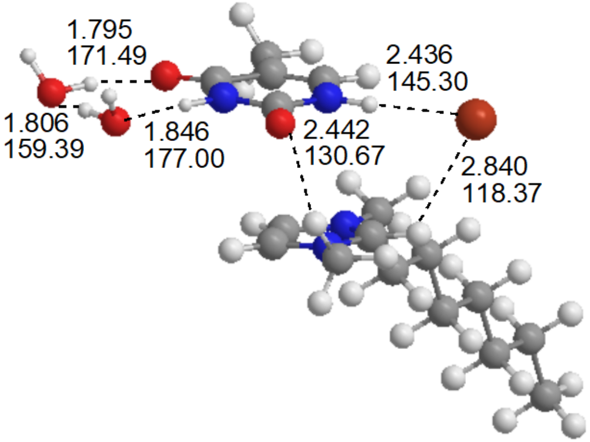

T-2H_2O-[C_8mim]$^+$-Br^--3 (5.77)

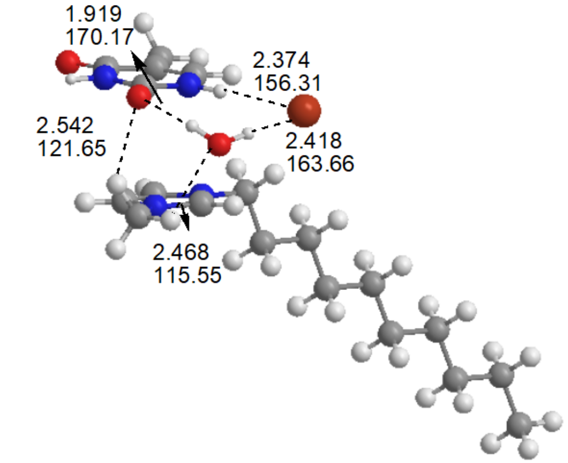

T-1H$_2$O-[C$_{10}$mim]$^+$-Br$^-$-1 (0.00)

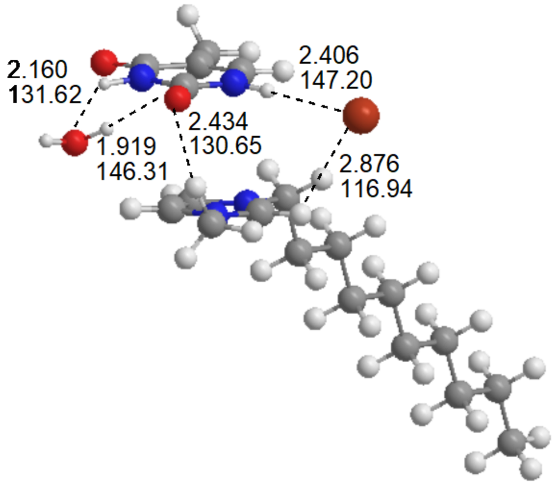

T-1H$_2$O-[C$_{10}$mim]$^+$-Br$^-$-2 (7.04)

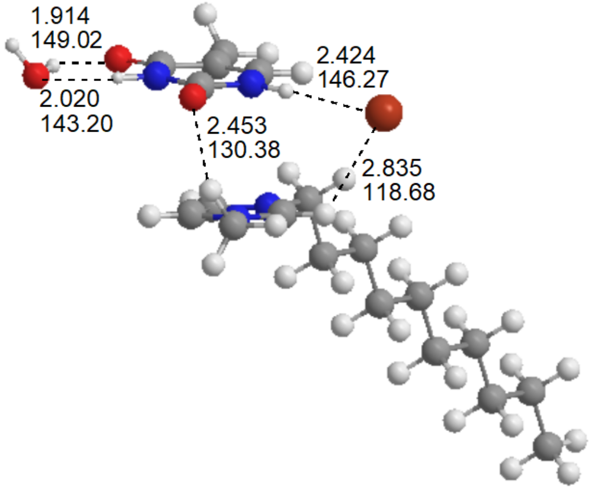

T-1H$_2$O-[C$_{10}$mim]$^+$-Br$^-$-3 (8.84)

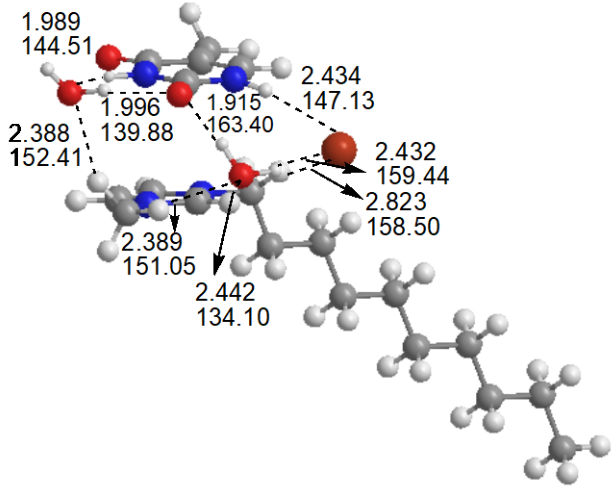

T-2H$_2$O-[C$_{10}$mim]$^+$-Br$^-$-1 (0.00)

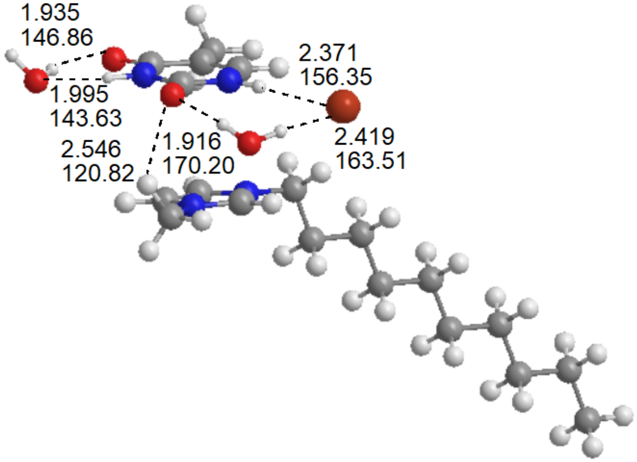

T-2H$_2$O-[C$_{10}$mim]$^+$-Br$^-$-2 (3.42)

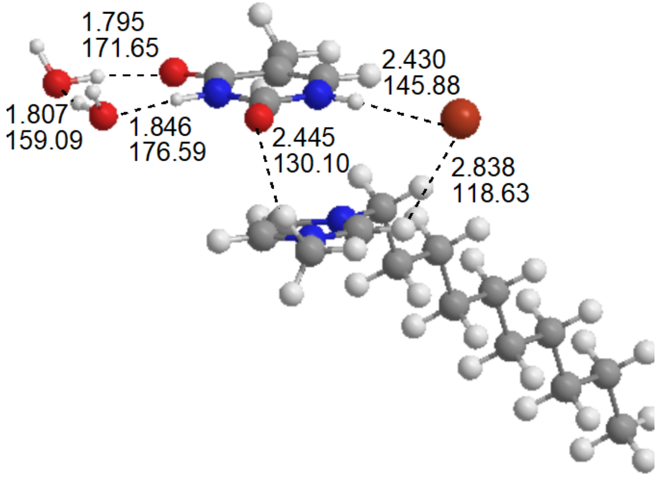

T-2H$_2$O-[C$_{10}$mim]$^+$-Br$^-$-3 (5.39)

Figure S1. Optimized molecular structures of micro-hydrated T-wH$_2$O-[C$_n$mim]$^+$-Br$^-$ (w = 1, 2 and n = 2, 4, 6, 8, 10) at the M06-2X/6-311++G(2*d*, *p*)/PCM/water level. The ZPVE-corrected stability decreases from left to right (in kJ/mol).

Occupied orbitals

T

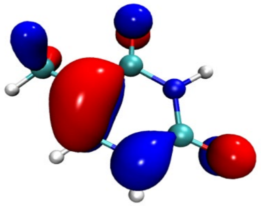

HOMO

T-4H_2O-Br^--1

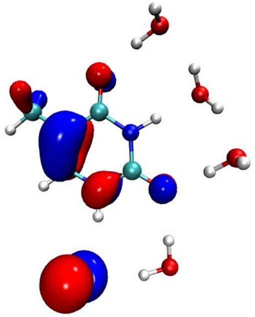

HOMO-2

T-3H_2O-$[C_2mim]^+$-Br^--1

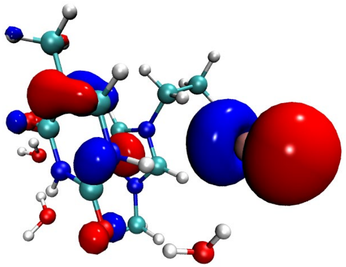

HOMO

T-2H_2O-$[C_2mim]^+$-2Br^--1

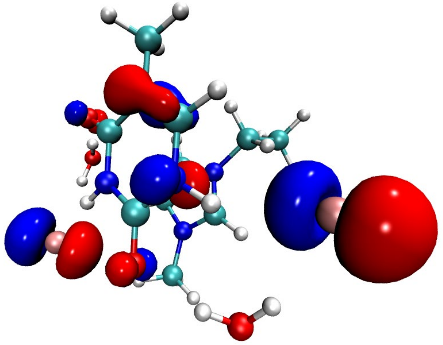

HOMO-1

T-1H_2O-2$[C_2mim]^+$-2Br^--1

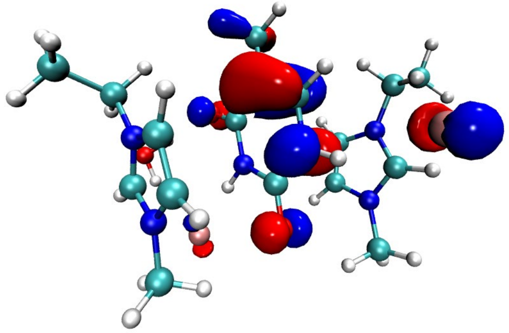

HOMO-3

continued

T-3H_2O-[C_4mim]$^+$-Br^--1

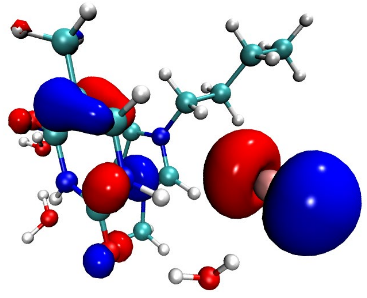

HOMO

T-2H_2O-[C_4mim]$^+$-2Br^--1

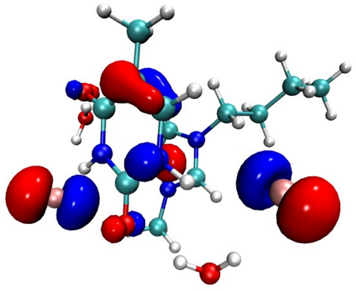

HOMO

T-1H_2O-2[C_4mim]$^+$-2Br^--1

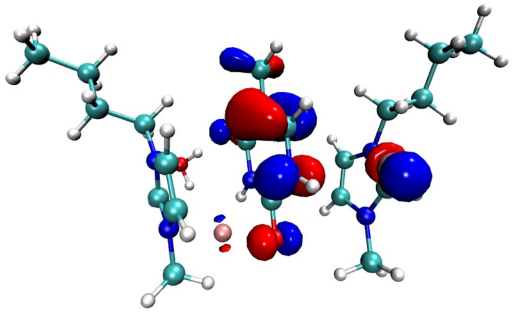

HOMO-3

T-3H_2O-[C_6mim]$^+$-Br^--1

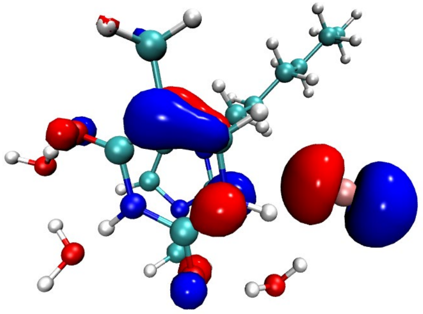

HOMO

T-2H_2O-[C_6mim]$^+$-2Br^--1

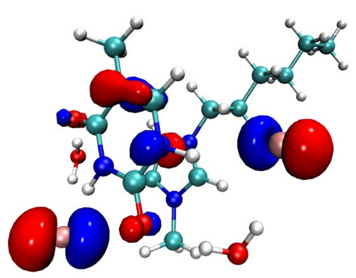

HOMO

T-1H_2O-2[C_6mim]$^+$-2Br^--1

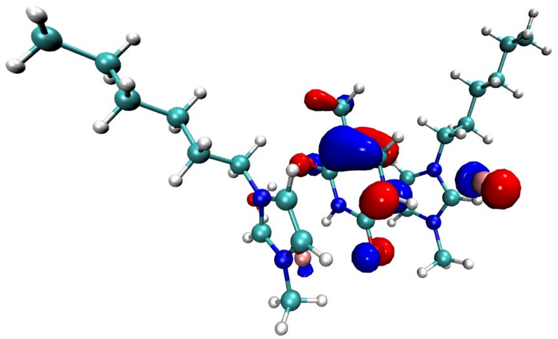

HOMO-3

continued

T-3H_2O-[C_8mim]$^+$-Br$^-$-1

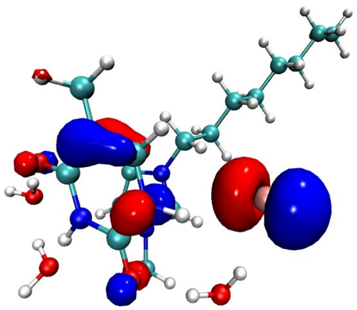

HOMO

T-2H_2O-[C_8mim]$^+$-2Br$^-$-1

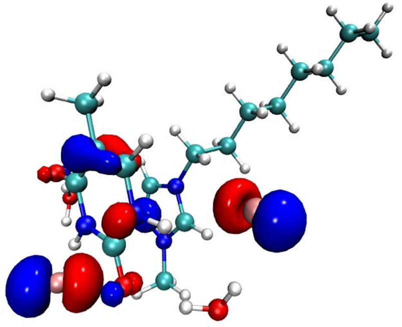

HOMO

T-1H_2O-2[C_8mim]$^+$-2Br$^-$-1

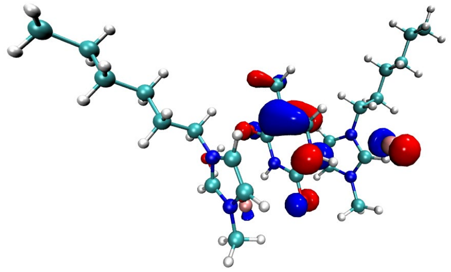

HOMO-3

T-3H_2O-[C_{10}mim]$^+$-Br$^-$-1

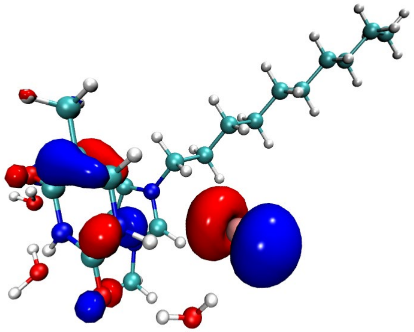

HOMO

T-2H_2O-[C_{10}mim]$^+$-2Br$^-$-1

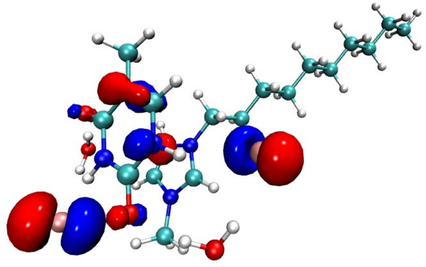

HOMO

T-1H_2O-2[C_{10}mim]$^+$-2Br$^-$-1

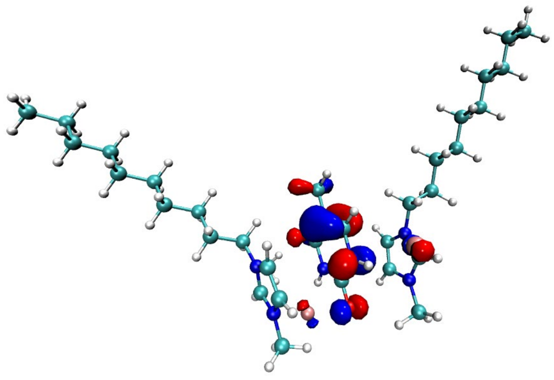

HOMO-3

Unoccupied orbitals

T

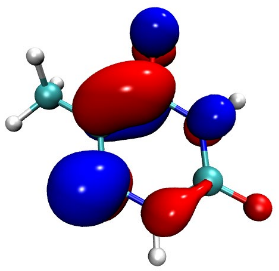

LUMO

T-4H_2O-Br^--1

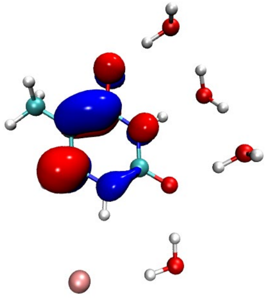

LUMO

T-3H_2O-$[C_2mim]^+$-Br^--1

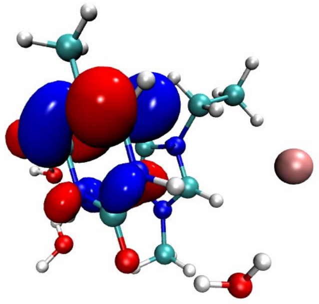

LUMO

T-2H_2O-$[C_2mim]^+$-2Br^--1

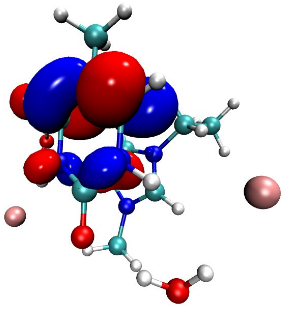

LUMO

T-1H_2O-2$[C_2mim]^+$-2Br^--1

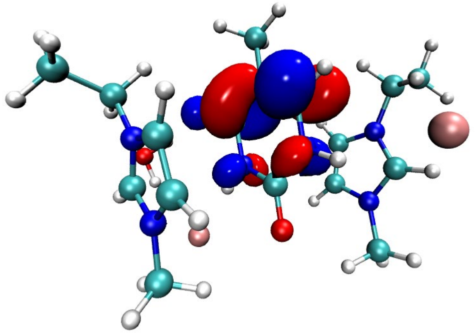

LUMO

continued

T-3H_2O-[C_4mim]$^+$-Br^--1

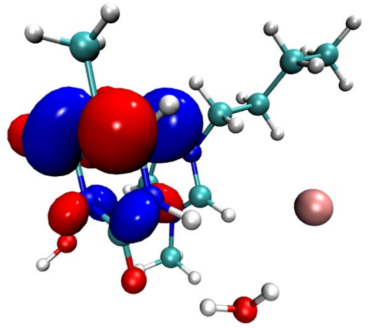

LUMO

T-2H_2O-[C_4mim]$^+$-2Br^--1

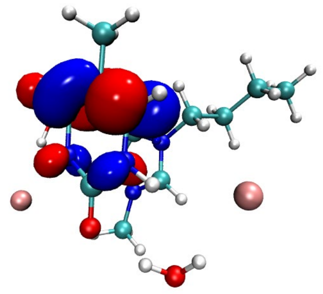

LUMO

T-1H_2O-2[C_4mim]$^+$-2Br^--1

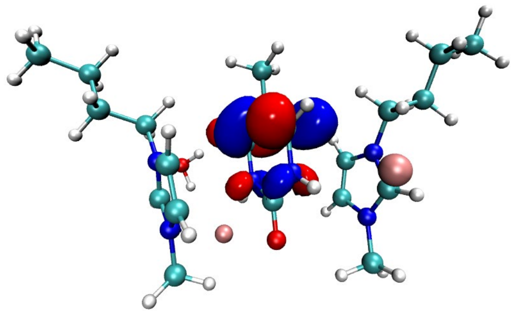

LUMO

T-3H_2O-[C_6mim]$^+$-Br^--1

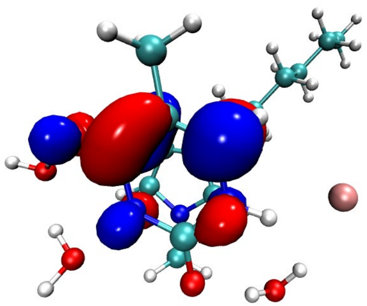

LUMO

T-2H_2O-[C_6mim]$^+$-2Br^--1

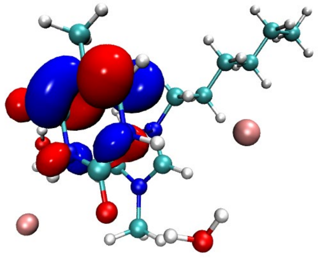

LUMO

T-1H_2O-2[C_6mim]$^+$-2Br^--1

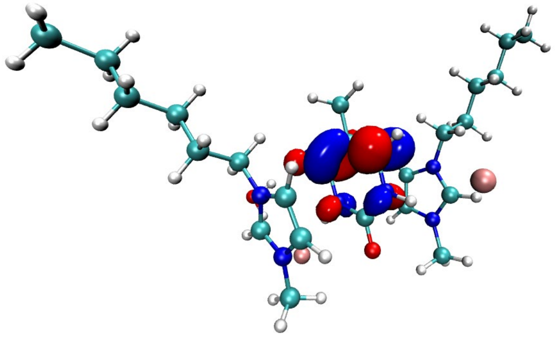

LUMO

continued

T-3H_2O-[C_8mim]$^+$-Br^--1

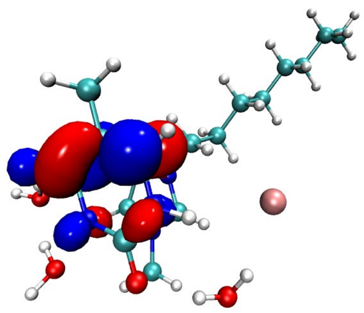

LUMO

T-2H_2O-[C_8mim]$^+$-2Br^--1

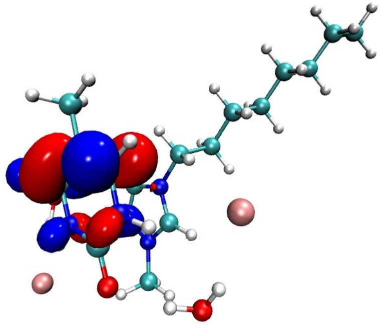

LUMO

T-1H_2O-2[C_8mim]$^+$-2Br^--1

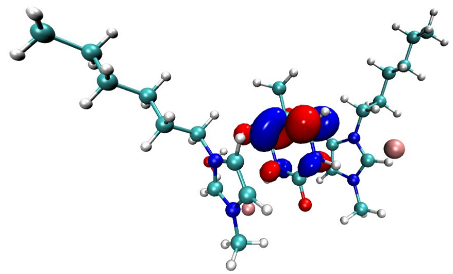

LUMO

T-3H_2O-[C_{10}mim]$^+$-Br^--1

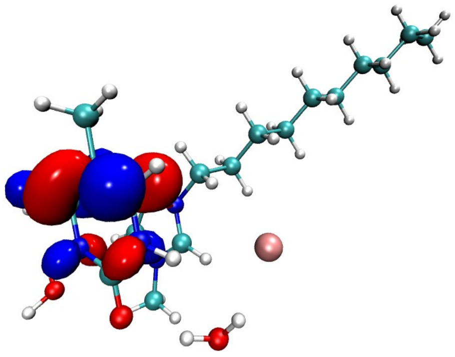

LUMO

T-2H_2O-[C_{10}mim]$^+$-2Br^--1

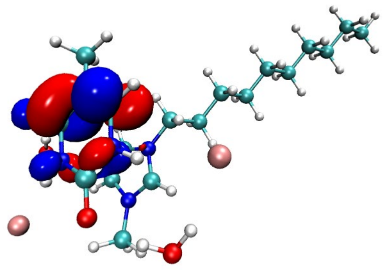

LUMO

T-1H_2O-2[C_{10}mim]$^+$-2Br^--1

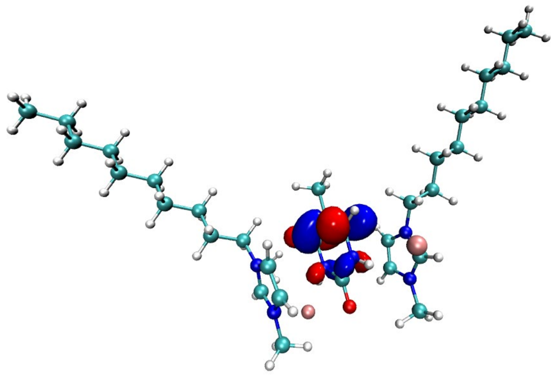

LUMO

Figure S2. The isosurface maps of the frontier molecular orbitals for the isolated T, micro-hydrated T-4H_2O-Br^--1, T-3H_2O-[$C_n$mim]$^+$-Br^--1, T-2H_2O-[$C_n$mim]$^+$-2Br^--1, and T-1H_2O-2[$C_n$mim]$^+$-2Br^--1 species calculated at the TD-M06-2X/6-311++G(2*d*, *p*)/PCM/water level (isovalues = 0.0 1 a.u.).
