# Supplementary material for: Density Functional Method Study on the Cooperativity of Intermolecular H-bonding and π-π+ Stacking Interactions in Thymine-[Cnmim]Br (n = 2, 4, 6, 8, 10) Microhydrates
Source: Molecules. 2022 Sep 22;27(19):6242. doi: 10.3390/molecules27196242 (PMC9572290; doi:10.3390/molecules27196242)
Supplement: Supplementary file 1 [file molecules-27-06242-s001.zip › Electronic Supplementary Information-Table S1-S8.pdf]

Density Functional Method Study on the Cooperativity of Intermolecular H-bonding and π-π[+] Stacking Interactions in Thymine-[$C_n$mim]Br (n = 2, 4, 6, 8, 10) Microhydrates

Yanni Wang[1], Chaowu Dai[1], Wei Huang[1], Tingting Ni[2], Jianping Cao[2], Jiangmei Pang[2], Huining Wei[2] and Chaojie Wang[2*]

[1]The Third Affiliated Hospital of Wenzhou Medical University; the Pharmacy Department of Ruian People's Hospital, Wenzhou 325000, Zhejiang, China.

[2]School of Pharmaceutical Sciences, Wenzhou Medical University, Wenzhou 325035, Zhejiang, China.

[*]Correspondence: chjwang@wmu.edu.cn

Contents

Table S1 Calculated geometrical parameters of the isolated thymine at the M06-2X/6-311++G(2*d*, *p*)/PCM/water level. Bond lengths in angstrom (Å) and angles in degree (°). 1

Table S2 Calculated energetic characteristics of the ionic T-*y*[$C_n$mim]$^+$-*x*Br^- (n = 2, 4, 6, 8, 10) for three stacking, perpendicular, and coplanar configurations at the M06-2X/6-311++G(2*d, p*)/PCM/water level (in kJ/mol). 3

Table S3 Calculated geometrical parameters of the ionic T-*y*[$C_n$mim]$^+$-*x*Br^- (n = 2, 4, 6, 8, 10) for the most stable stacking configuration at the M06-2X/6-311++G(2*d, p*)/PCM/water level. Bond lengths in angstrom (Å). 4

Table S4 Calculated energetic characteristics of the stepwise microhydrated T-*w*H_2O-*y*[$C_n$mim]$^+$-*x*Br^- (*w* = 1, 2, 3, 4, 5 and n = 2, 4, 6, 8, 10) for each most stable configuration at the M06-2X/6-311++G(2*d*, *p*)/PCM/water level (in kJ/mol). 5

Table S5 Calculated geometrical parameters of the microhydrated T-*w*H_2O-*y*[$C_n$mim]$^+$-*x*Br^- (*w* = 1, 2, 3, 4, 5 and n = 2, 4, 6, 8, 10) for the most stable configuration at the M06-2X/6-311++G(2*d*, *p*)/PCM/water level. Bond lengths in angstrom (Å) 6

Table S6 Calculated wavenumbers (Wave, cm^{-1}), intensities (Intens., km/mol) of vibrational modes and the corresponding main contributions (Contri., %) to the absorption maxima (Absorp., cm^{-1}) for the isolated thymine 7

Table S7 Calculated wavenumbers (Wave, cm^{-1}), intensities (Intens., km/mol) of vibrational modes and the corresponding main contributions (Contri., %) to the absorption maxima (Absorp., cm^{-1}) located within 3700-3300 cm^{-1} and 1800-1700 cm^{-1} for the thymine in the most stable T-4H_2O-Br^--1, T-3H_2O-[$C_n$mim]$^+$-Br^--1, T-2H_2O-[$C_n$mim]$^+$-2Br^--1, and T-1H_2O-2[$C_n$mim]$^+$-2Br^--1 micro-hydrates. 9

Table S8 NPA charge and charge transfer in the most stable T-4H_2O-Br^--1, T-3H_2O-[$C_n$mim]$^+$-Br^--1, T-2H_2O-[$C_n$mim]$^+$-2Br^--1, and T-1H_2O-2[$C_n$mim]$^+$-2Br^--1 micro-hydrates with respect to the corresponding isolated fragment, respectively (in a.u.). 12

Table S1. Calculated geometrical parameters of the isolated thymine at the M06-2X/6-311++G(2*d*, *p*)/PCM/water level. Bond lengths in angstrom (Å) and angles in degree (°).

Bond distance (Å)	Exp.[a]	Calc.[a1]	Calc.[a2]	Calc.[c]	Bond angles (°)	Exp.[a]	Calc.[a1]	Calc.[a2]	Calc.[c]
**N1-C2**	1.358	1.367	1.407	1.379	**C2-N1-C6**	122.5	123.5	124.2	123.6
**C2-N3**	1.361	1.369	1.400	1.379	**N1-C2-N3**	115.5	113.2	111.9	113.0
**N3-C4**	1.401	1.387	1.426	1.398	**C2-N3-C4**	126.3	127.8	128.5	127.7
**C4-C5**	1.453	1.471	1.476	1.466	**N3-C4-C5**	115.1	114.8	114.3	114.9
**C5=C6**	1.343	1.328	1.365	1.340	**C4-C5=C6**	118.4	117.7	118.4	118.0
**C6-N1**	1.384	1.375	1.389	1.377	**N1-C6=C5**	122.3	123.0	122.6	122.8
**C2=O7**	1.244	1.192	1.23	1.207	**N1-C2=O7**	122.4	123.3	123.2	123.2
**C4=O8**	1.225	1.193	1.235	1.209	**N3-C4=O8**	119.2	120.6	120.3	120.5
**C5-C9**	1.502	1.508	1.511	1.495					
**N1-H**		0.995	1.019	1.008					
**N3-H**		0.998	1.022	1.012					
**C6-H**		1.073	1.093	1.083					

Exp.[a] observed values cited from X-ray data of thymine from ref. [75]
Cal.[a1] and Cal.[a2] predicted values of thymine based on HF/4-31G** and B3LYP/6-31G** levels are both from ref. [75]
Cal.[c] this work

Table S2. Calculated energetic characteristics of the ionic T-*y*[$C_n$mim]$^+$-*x*Br^- (n = 2, 4, 6, 8, 10) for three stacking, perpendicular, and coplanar configurations at the M06-2X/6-311++G(2*d, p*)/PCM/water level (in kJ/mol).

Complexes	Config.	E_B	E_D^T	E_{BSSE}	Complexes	Config.	E_B	E_D^T	E_{BSSE}	Complexes	Config.	E_B	E_D^T	E_{BSSE}
**T-Br⁻-1**	front	-22.16	1.11	1.13										
**T-[C_2mim]$^+$-1**	S1	-16.49	0.35	3.70	**T-[C_2mim]$^+$-5**	P1	-13.12	0.08	1.54	**T-[C_2mim]$^+$-9**	H1	-12.00	0.04	1.28
**T-[C_4mim]$^+$-1**	S1	-15.35	0.31	3.79	**T-[C_4mim]$^+$-5**	P1	-11.24	0.07	1.60	**T-[C_4mim]$^+$-9**	H1	-11.49	0.04	1.29
**T-[C_6mim]$^+$-1**	S1	-16.86	0.28	3.87	**T-[C_6mim]$^+$-5**	P1	-13.33	0.10	1.65	**T-[C_6mim]$^+$-9**	H1	-12.62	0.05	1.30
**T-[C_8mim]$^+$-1**	S1	-16.66	0.29	3.91	**T-[C_8mim]$^+$-5**	P1	-13.06	0.08	1.66	**T-[C_8mim]$^+$-9**	H1	-12.00	0.04	1.31
**T-[C_{10}mim]$^+$-1**	S1	-16.28	0.29	3.90	**T-[C_{10}mim]$^+$-5**	P1	-11.64	0.08	1.65	**T-[C_{10}mim]$^+$-9**	H1	-10.62	0.05	1.28
**T-[C_2mim]$^+$-Br^--1**	S1	-54.41	1.32	5.22	**T-[C_2mim]$^+$-Br^--5**	P1	-50.46	2.42	1.28	**T-[C_2mim]$^+$-Br^--9**	H1	-46.33	2.31	0.88
**T-[C_4mim]$^+$-Br^--1**	S1	-53.16	1.32	5.26	**T-[C_4mim]$^+$-Br^--5**	P1	-49.37	2.39	1.29	**T-[C_4mim]$^+$-Br^--9**	H1	-47.55	2.25	0.91
**T-[C_6mim]$^+$-Br^--1**	S1	-54.69	1.28	5.28	**T-[C_6mim]$^+$-Br^--5**	P1	-50.54	2.41	1.33	**T-[C_6mim]$^+$-Br^--9**	H1	-48.54	2.24	0.92
**T-[C_8mim]$^+$-Br^--1**	S1	-54.61	1.30	5.32	**T-[C_8mim]$^+$-Br^--5**	P1	-49.90	2.44	1.33	**T-[C_8mim]$^+$-Br^--9**	H1	-48.96	2.24	0.91
**T-[C_{10}mim]$^+$-Br^--1**	S1	-53.40	1.33	5.33	**T-[C_{10}mim]$^+$-Br^--5**	P1	-48.95	2.45	1.32	**T-[C_{10}mim]$^+$-Br^--9**	H1	-47.75	2.20	0.91
**T-[C_2mim]$^+$-2Br^--1**	S	-79.08	2.09	6.99	**T-[C_2mim]$^+$-2Br^--5**	P	-66.65	2.22	5.07	**T-[C_2mim]$^+$-2Br^--9**	H	-63.77	1.94	4.51
**T-[C_4mim]$^+$-2Br^--1**	S	-74.42	3.17	6.51	**T-[C_4mim]$^+$-2Br^--5**	P	-65.63	1.97	3.53	**T-[C_4mim]$^+$-2Br^--9**	H	-64.50	1.56	3.29
**T-[C_6mim]$^+$-2Br^--1**	S	-75.04	3.14	6.53	**T-[C_6mim]$^+$-2Br^--5**	P	-67.26	1.96	3.55	**T-[C_6mim]$^+$-2Br^--9**	H	-66.24	1.54	3.28
**T-[C_8mim]$^+$-2Br^--1**	S	-79.67	2.93	7.06	**T-[C_8mim]$^+$-2Br^--5**	P	-64.58	1.97	3.59	**T-[C_8mim]$^+$-2Br^--9**	H	-65.53	1.57	3.28
**T-[C_{10}mim]$^+$-2Br^--1**	S	-74.29	3.09	6.65	**T-[C_{10}mim]$^+$-2Br^--5**	P	-64.85	1.96	3.56	**T-[C_{10}mim]$^+$-2Br^--9**	H	-65.07	1.60	3.24
**T-2[C_2mim]$^+$-2Br^--1**	S	-109.47	2.09	11.17	**T-2[C_2mim]$^+$-2Br^--5**	P	-96.88	2.22	5.07	**T-2[C_2mim]$^+$-2Br^--9**	H	-90.75	1.94	4.51
**T-2[C_4mim]$^+$-2Br^--1**	S	-107.80	2.09	11.15	**T-2[C_4mim]$^+$-2Br^--5**	P	-94.79	2.41	4.98	**T-2[C_4mim]$^+$-2Br^--9**	H	-92.70	2.00	4.29
**T-2[C_6mim]$^+$-2Br^--1**	S	-109.91	2.11	11.13	**T-2[C_6mim]$^+$-2Br^--5**	P	-96.87	2.40	5.04	**T-2[C_6mim]$^+$-2Br^--9**	H	-95.68	1.94	4.31
**T-2[C_8mim]$^+$-2Br^--1**	S	-109.71	2.10	11.25	**T-2[C_8mim]$^+$-2Br^--5**	P	-95.67	2.33	5.08	**T-2[C_8mim]$^+$-2Br^--9**	H	-95.01	1.99	4.34
**T-2[C_{10}mim]$^+$-2Br^--1**	S	-107.61	2.10	11.27	**T-2[C_{10}mim]$^+$-2Br^--5**	P	-93.96	2.40	5.00	**T-2[C_{10}mim]$^+$-2Br^--9**	H	-92.95	1.94	4.36

Table S3. Calculated geometrical parameters of the ionic T-*y*[$C_n$mim]$^+$-*x*Br^- (n = 2, 4, 6, 8, 10) for the most stable stacking configuration at the M06-2X/6-311++G(2*d, p*)/PCM/water level. Bond lengths in angstrom (Å).

**Geometrics**	**T**					**[$C_n$mim]$^+$**			**T-[$C_n$mim]$^+$**
	**N1-H**	**C2=O7**	**N3-H**	**C4=O8**	**C6-H**	**$^+$C(2)-H**	**$^+$C(6)-H**	**$^+$C(7)-H**	**$\pi\cdots\pi^+$**
**Monomer**	1.010	1.216	1.013	1.219	1.082	1.078	1.086	1.088	-
**T-Br^--1**	1.025	1.219	1.013	1.221	1.083	-	-	-	
**T-[C_2mim]$^+$-1**	1.011	1.217	1.013	1.217	1.083	1.078	1.086	1.088	3.411
**T-[C_4mim]$^+$-1**	1.011	1.217	1.013	1.217	1.083	1.078	1.086	1.090	3.420
**T-[C_6mim]$^+$-1**	1.011	1.217	1.013	1.217	1.083	1.078	1.086	1.090	3.399
**T-[C_8mim]$^+$-1**	1.011	1.217	1.013	1.217	1.083	1.078	1.086	1.090	3.383
**T-[C_{10}mim]$^+$-1**	1.011	1.217	1.013	1.217	1.082	1.078	1.086	1.090	3.413
**T-[C_2mim]$^+$-Br^--1**	1.022	1.219	1.013	1.219	1.084	1.078	1.076	1.090	3.446
**T-[C_4mim]$^+$-Br^--1**	1.022	1.219	1.013	1.219	1.084	1.078	1.076	1.092	3.424
**T-[C_6mim]$^+$-Br^--1**	1.022	1.219	1.013	1.219	1.084	1.078	1.076	1.092	3.449
**T-[C_8mim]$^+$-Br^--1**	1.022	1.219	1.013	1.219	1.084	1.078	1.076	1.092	3.395
**T-[C_{10}mim]$^+$-Br^--1**	1.022	1.219	1.013	1.219	1.084	1.078	1.076	1.091	3.412
**T-[C_2mim]$^+$-2Br^--1**	1.021	1.220	1.027	1.221	1.083	1.083	1.086	1.091	3.306
**T-[C_4mim]$^+$-2Br^--1**	1.023	1.220	1.024	1.222	1.084	1.078	1.086	1.091	3.347
**T-[C_6mim]$^+$-2Br^--1**	1.023	1.221	1.024	1.222	1.084	1.078	1.086	1.091	3.369
**T-[C_8mim]$^+$-2Br^--1**	1.021	1.220	1.027	1.221	1.083	1.082	1.086	1.091	3.331
**T-[C_{10}mim]$^+$-2Br^--1**	1.023	1.221	1.024	1.222	1.084	1.078	1.086	1.091	3.336
**T-2[C_2mim]$^+$-2Br^--1**	1.022	1.222	1.028	1.222	1.084	1.078	1.087	1.090	3.358
**T-2[C_4mim]$^+$-2Br^--1**	1.023	1.222	1.028	1.222	1.084	1.078	1.086	1.091	3.397
**T-2[C_6mim]$^+$-2Br^--1**	1.022	1.222	1.028	1.222	1.084	1.078	1.086	1.091	3.410
**T-2[C_8mim]$^+$-2Br^--1**	1.022	1.222	1.028	1.222	1.084	1.078	1.086	1.091	3.419
**T-2[C_{10}mim]$^+$-2Br^--1**	1.022	1.222	1.028	1.222	1.084	1.078	1.086	1.091	3.388

Table S4. Calculated energetic characteristics of the stepwise micro-hydrated T-*w*H_2O-*y*$[C_nmim]^+$-*x*Br^- (w = 1, 2, 3, 4, 5 and n = 2, 4, 6, 8, 10) for each most stable configuration at the M06-2X/6-311++G(2*d*, *p*)/PCM/water level (in kJ/mol).

	Complexes	E_B	E_D^T	E_{BSSE}		Complexes	E_B	E_D^T	E_{BSSE}
**T-*w*H_2O-1**	**T-1H_2O-1**	-15.45	0.50	2.83	**T-*w*H_2O-Br^--1**	**T-1H_2O-Br^--1**	-43.39	1.34	2.43
	**T-2H_2O-1**	-28.27	1.67	4.14		**T-2H_2O-Br^--1**	-58.93	2.06	5.38
	**T-3H_2O-1**	-43.79	2.37	6.88		**T-3H_2O-Br^--1**	-81.29	2.80	6.09
	**T-4H_2O-1**	-55.32	3.41	8.52		**T-4H_2O-Br^--1**	-102.74	4.89	7.74
	**T-5H_2O-1**	-97.39	5.44	9.12		-	-	-	-
**T-*w*H_2O-$[C_nmim]^+Br^-$-1**	**T-1H_2O-$[C_2mim]^+$-Br^--1**	-79.13	1.69	6.41	**T-*w*-H_2O-$[C_nmim]^+$-2Br^--1**	**T-1H_2O-$[C_2mim]^+$-2Br^--1**	-106.12	3.12	8.37
	**T-1H_2O-$[C_4mim]^+$-Br^--1**	-78.69	1.73	6.52		**T-1H_2O-$[C_4mim]^+$-2Br^--1**	-106.04	3.13	8.28
	**T-1H_2O-$[C_6mim]^+$-Br^--1**	-80.12	1.68	6.60		**T-1H_2O-$[C_6mim]^+$-2Br^--1**	-107.90	3.10	8.41
	**T-1H_2O-$[C_8mim]^+$-Br^--1**	-79.65	1.73	6.65		**T-1H_2O-$[C_8mim]^+$-2Br^--1**	-106.90	3.12	8.46
	**T-1H_2O-$[C_{10}mim]^+$-Br^--1**	-79.00	1.74	6.66		**T-1H_2O-$[C_{10}mim]^+$-2Br^--1**	-106.17	3.15	8.48
	**T-2H_2O-$[C_2mim]^+$-Br^--1**	-96.55	3.20	9.34		**T-2H_2O-$[C_2mim]^+$-2Br^--1**	-131.01	3.35	9.59
	**T-2H_2O-$[C_4mim]^+$-Br^--1**	-94.36	2.42	9.07		**T-2H_2O-$[C_4mim]^+$-2Br^--1**	-130.93	3.28	9.69
	**T-2H_2O-$[C_6mim]^+$-Br^--1**	-97.94	3.15	9.38		**T-2H_2O-$[C_6mim]^+$-2Br^--1**	-132.30	3.31	9.69
	**T-2H_2O-$[C_8mim]^+$-Br^--1**	-97.86	3.15	9.40		**T-2H_2O-$[C_8mim]^+$-2Br^--1**	-132.97	3.28	9.62
	**T-2H_2O-$[C_{10}mim]^+$-Br^--1**	-93.82	2.41	9.08		**T-2H_2O-$[C_{10}mim]^+$-2Br^--1**	-131.32	3.30	9.75
	**T-3H_2O-$[C_2mim]^+$-Br^--1**	-126.09	4.00	10.37	**T-*w*-H_2O-2$[C_nmim]^+$-2Br^--1**	**T-1H_2O-2$[C_2mim]^+$-2Br^--1**	-137.46	2.89	12.33
	**T-3H_2O-$[C_4mim]^+$-Br^--1**	-125.90	4.00	10.40		**T-1H_2O-2$[C_4mim]^+$-2Br^--1**	-134.71	2.81	12.41
	**T-3H_2O-$[C_6mim]^+$-Br^--1**	-127.37	3.97	10.48		**T-1H_2O-2$[C_6mim]^+$-2Br^--1**	-137.63	2.83	12.40
	**T-3H_2O-$[C_8mim]^+$-Br^--1**	-126.52	4.00	10.36		**T-1H_2O-2$[C_8mim]^+$-2Br^--1**	-136.71	2.86	12.46
	**T-3H_2O-$[C_{10}mim]^+$-Br^--1**	-126.32	3.98	10.45		**T-1H_2O-2$[C_{10}mim]^+$-2Br^--1**	-136.29	2.85	12.48

Table S5. Calculated geometrical parameters of the micro-hydrated T-wH$_2$O-y[C$_n$mim]$^+$-xBr$^-$ (w = 1, 2, 3, 4, 5 and n = 2, 4, 6, 8, 10) for the most stable configuration at the M06-2X/6-311++G(2d, p)/PCM/water level. Bond lengths in angstrom (Å)

Geometrics	T					[C$_n$mim]$^+$			T-[C$_n$mim]$^+$
	N1-H	C2=O7	N3-H	C4=O8	C6-H	$^+$C(2)-H	$^+$C(6)-H	$^+$C(7)-H	$\pi\cdots\pi^+$
**Monomer**	1.010	1.216	1.013	1.219	1.082	1.08	1.09	1.09	-
**T-5H$_2$O-1**	1.030	1.237	1.037	1.228	1.082	-	-	-	-
**T-4H$_2$O-Br$^-$-1**	1.026	1.234	1.036	1.229	1.083	-	-	-	-
**T-3H$_2$O-[C$_2$mim]$^+$-Br$^-$-1**	1.021	1.225	1.030	1.228	1.083	1.081	1.086	1.091	3.320
**T-3H$_2$O-[C$_4$mim]$^+$-Br$^-$-1**	1.021	1.225	1.030	1.228	1.083	1.081	1.086	1.091	3.312
**T-3H$_2$O-[C$_6$mim]$^+$-Br$^-$-1**	1.021	1.225	1.030	1.228	1.083	1.081	1.087	1.091	3.327
**T-3H$_2$O-[C$_8$mim]$^+$-Br$^-$-1**	1.021	1.225	1.030	1.228	1.083	1.081	1.086	1.091	3.322
**T-3H$_2$O-[C$_{10}$mim]$^+$-Br$^-$-1**	1.021	1.225	1.030	1.228	1.083	1.081	1.086	1.091	3.306
**T-2H$_2$O-[C$_2$mim]$^+$-2Br$^-$-1**	1.020	1.225	1.029	1.225	1.083	1.081	1.086	1.091	3.306
**T-2H$_2$O-[C$_4$mim]$^+$-2Br$^-$-1**	1.020	1.225	1.029	1.225	1.083	1.081	1.087	1.092	3.318
**T-2H$_2$O-[C$_6$mim]$^+$-2Br$^-$-1**	1.020	1.225	1.029	1.225	1.083	1.081	1.086	1.091	3.297
**T-2H$_2$O-[C$_8$mim]$^+$-2Br$^-$-1**	1.020	1.225	1.029	1.225	1.083	1.081	1.086	1.092	3.322
**T-2H$_2$O-[C$_{10}$mim]$^+$-2Br$^-$-1**	1.020	1.225	1.029	1.225	1.083	1.081	1.086	1.091	3.304
**T-1H$_2$O-2[C$_2$mim]$^+$-2Br$^-$-1**	1.023	1.220	1.029	1.228	1.084	1.079	1.086	1.088	3.405
**T-1H$_2$O-2[C$_4$mim]$^+$-2Br$^-$-1**	1.023	1.220	1.029	1.228	1.084	1.078	1.086	1.089	3.393
**T-1H$_2$O-2[C$_6$mim]$^+$-2Br$^-$-1**	1.023	1.220	1.029	1.228	1.084	1.078	1.085	1.089	3.386
**T-1H$_2$O-2[C$_8$mim]$^+$-2Br$^-$-1**	1.023	1.220	1.029	1.228	1.084	1.079	1.085	1.089	3.385
**T-1H$_2$O-2[C$_{10}$mim]$^+$-2Br$^-$-1**	1.023	1.220	1.029	1.228	1.084	1.079	1.086	1.089	3.402

Table S6. Calculated wavenumbers (Wave, cm^{-1}), intensities (Intens., km/mol) of vibrational modes and the corresponding main contributions (Contri., %) to the absorption maxima (Absorp., cm^{-1}) for the isolated thymine

Max.	Mode.	Contri.	Wave	Intens.	Assign.
3636	**v1**	96	3636	233	**υN1-H**
3599	**v2**	89	3599	135	**υN3-H**
3244	v3	79	3244	3	υC6-H
3150	v4	90	3150	12	$υ^{as}$CH3
3114	v5	84	3114	10	$υ^{as}$CH3
3054	v6	97	3054	23	$υ^{s}$CH3
1774	**v7**	90	1774	871	**υC2=O7 +** ρN1-H
1734	**v8**	44	1735	617	**υC4=O8** + ρN3-H
1723	**v9**	84	1723	1629	**υC5=C6** + ρN3-H + ρC6-H
1523	v10	96	1523	235	βN1-H
1486	v11	30	1487	1	$δ^{as}$CH3
1470	v12	21	1471	13	$δ^{as}$CH3
1451	v13	92	1451	153	ρN1-H
	v14		1422	5	ρN3-H + $δ^{s}$CH3
1420	v15	21	1420	7	$δ^{s}$CH3 + ρN3-H
1388	v16	47	1388	9	β C6-H
1251	v17	13	1251	4	υC5-CH3
1215	v18	99	1215	342	υ ring
1172	v19	51	1173	20	υ ring
1076	v20	38	1077	3	ρCH3
1034	v21	69	1034	9	ρCH3
991	v22	84	991	21	ρCH3
956	v23	84	957	20	γC6-H + γCH3
818	v24	64	818	17	α ring
790	v25	78	790	49	γC4=O8

Table S6. (*continued*)

771	v26	71	771	39	γC2=O7
752	v27	36	752	9	υC4-C5
671	v28	98	671	138	γN3-H
616	v29	18	616	2	γN3-H
559	v30	86	559	93	α ring
	v31		558	13	γN1-H

v_{as}: asymmetric stretching; v_s: symmetric stretching; α: angle bending; β: in-plane bending; γ: out-of-plane bending; δ: deformation; ρ: rocking

Table S7. Calculated wavenumbers (Wave, cm^{-1}), intensities (Intens., km/mol) of vibrational modes and the corresponding main contributions (Contri., %) to the absorption maxima (Absorp., cm^{-1}) located within 3700-3300 cm^{-1} and 1800-1700 cm^{-1} for the thymine in the most stable T-$4H_2O$-Br^--1, T-$3H_2O$-$[C_nmim]^+$-Br^--1, T-$2H_2O$-$[C_nmim]^+$-$2Br^-$-1, and T-$1H_2O$-$2[C_nmim]^+$-$2Br^-$-1 microhydrates.

Absorp.	Mode	Contri.	Assign.	Wave	Intens.	Absorp.	Mode	Contri.	Assign.	Wave	Intens.
**T-$4H_2O$-Br^--1**						**T-$3H_2O$-$[C_2mim]^+$-Br^--1**					
3336	v9	100	νN1-H	3336	1833	3436	v7	100	νN1-H	3436	1055
3125	v13	99	νN3-H	3125	1622	3277	v10	99	νN3-H	3277	1098
1722	v16	83	νC2=O7 + ρN3-H + ρC6-H+ ρN1-H	1722	882	1747	v24	98	νC2=O7+ ρN1-H + ρN3-H	1748	997
1705	v15	97	νC4=O8 + ρN3-H	1705	1677	1713	v26	98	νC4=O8 + ρN3-H	1713	1246
	v17		νC5=C6 + ρN1-H + ρC6-H + ρN3-H	1729	332		v25		νC5=C6 + ρC6-H + ρN1-H	1726	110
**T-$3H_2O$-$[C_4mim]^+$-Br^--1**						**T-$3H_2O$-$[C_6mim]^+$-Br^--1**					
3453	v7	100	νN1-H	3453	975	3453	v7	100	νN1-H	3453	956
3278	v10	99	νN3-H	3278	1094	3281	v10	100	νN3-H	3281	1085
1749	v28	98	νC2=O7+ ρN1-H + ρN3-H	1748	998	1749	v32	98	νC2=O7+ ρN1-H + ρN3-H	1749	1006
1713	v30	98	νC4=O8 + ρN3-H	1713	1263	1713	v34	98	νC4=O8 + ρN3-H	1712	1260
	v29		νC5=C6 + ρC6-H + ρN1-H	1726	106		v33		νC5=C6 + ρC6-H + ρN1-H	1726	100
**T-$3H_2O$-$[C_8mim]^+$-Br^--1**						**T-$3H_2O$-$[C_{10}mim]^+$-Br^--1**					
3444	v7	100	νN1-H	3443	1016	3444	v7	100	νN1-H	3444	1008
3280	v10	99	νN3-H	3279	1099	3281	v10	99	νN3-H	3280	1096
1749	v36	98	νC2=O7+ ρN1-H +ρN3-H	1749	990	1749	v40	98	νC2=O7+ρN1-H + ρN3-H	1749	993
1713	v38	98	νC4=O8 + ρN3-H	1712	1254	1713	v42	98	νC4=O8 + ρN3-H	1712	1251
	v37		νC5=C6 + ρC6-H + ρN1-H	1726	107		v41		νC5=C6 + ρC6-H + ρN1-H	1726	112

Table S7. (*continued*)

**T-2H_2O-[C_2mim]$^+$-2Br^--1**						**T-1H_2O-2[C_2mim]$^+$-2Br^--1**					
3453	5	100	νN1-H	3453	968	3388	v3	100	νN1-H	3388	1306
3320	7	98	νN3-H	3319	1414	3310	v5	95	νN3-H	3311	1488
1747	12	98	νC2=O7 + ρN1-H + ρN3-H	1747	806	1758	v30	99	νC2=O7 + ρN1-H + ρN3-H	1757	931
1727	14	43	νC4=O8 + ρN3-H	1708	1581	1702	v32	99	νC4=O8 + ρN3-H	1702	1484
1707	13	99	νC5=C6 + ρC6-H + ρN1-H	1728	56	1722	v31	42	νC5=C6 + ρC6-H + ρN1-H	1722	65
**T-2H_2O-[C_4mim]$^+$-2Br^--1**						**T-1H_2O-2[C_4mim]$^+$-2Br^--1**					
3461	5	100	νN1-H	3462	902	3401	v3	100	νN1-H	3402	1246
3322	7	86	νN3-H	3323	1251	3312	v5	97	νN3-H	3311	1514
1747	15	97	νC2=O7 + ρN1-H + ρN3-H	1747	801	1758	v38	99	νC2=O7 + ρN1-H + ρN3-H	1758	936
1709	17	99	νC4=O8 + ρN3-H	1709	1572	1722	v40	50	νC4=O8 + ρN3-H	1703	1482
1729	16	39	νC5=C6 + ρC6-H + νC4=O8 + ρN1-H	1729	59	1703	v39	99	νC5=C6 + ρC6-H + νC4=O8 + ρN1-H	1723	78
**T-2H_2O-[C_6mim]$^+$-2Br^--1**						**T-1H_2O-2[C_6mim]$^+$-2Br^--1**					
3460	5	100	νN1-H	3460	919	3400	v3	100	νN1-H	3399	1245
3320	7	99	νN3-H	3319	1419	3314	v5	95	νN3-H	3315	1497
1749	30	98	νC2=O7 + ρN1-H + ρN3-H	1748	805	1758	v48	99	νC2=O7 + ρN1-H + ρN3-H	1758	942

1710	32	99	νC4=O8 + ρN3-H	1709	1544	1721	v50	46	νC4=O8 + ρN3-H	1703	1479
1727	31	48	νC5=C6 + ρC6-H +ρN1-H	1728	50	1702	v49	99	νC5=C6 + ρC6-H +ρN1-H	1721	73
**T-2H_2O-[C_8mim]$^+$-2Br$^-$-1**						**T-1H_2O-2[C_8mim]$^+$-2Br$^-$-1**					
3450	5	100	νN1-H	3451	956	3394	v3	100	νN1-H	3394	1273
3317	7	99	νN3-H	3317	1433	3310	v5	91	νN3-H	3310	1437
1749	34	98	νC2=O7 + ρN1-H + ρN3-H	1749	807	1758	v54	99	νC2=O7 + ρN1-H + ρN3-H	1758	936
1709	36	99	νC4=O8 + ρN3-H	1709	1522	1703	v51	99	νC4=O8 + ρN3-H	1703	1480
1729	35	39	νC5=C6 + ρC6-H + ρN1-H	1729	62	1721	v50	48	νC5=C6 + ρC6-H +ρN1-H	1722	70
**T-2H_2O-[C_{10}mim]$^+$-2Br$^-$-1**						**T-1H_2O-2[C_{10}mim]$^+$-2Br$^-$-1**					
3454	5	100	νN1-H	3455	914	3396	v3	100	νN1-H	310	1257
3318	7	98	νN3-H	3318	1407	3313	v5	84	νN3-H	3313	1330
1749	38	98	νC2=O7 + ρN1-H + ρN3-H	1748	800	1758	v63	99	νC2=O7 + ρN1-H + ρN3-H	1757	934
1727	40	46	νC4=O8 + ρN3-H	1709	1540	1703	v65	99	νC4=O8 + ρN3-H	1703	1488
1709	39	99	νC5=C6 + ρC6-H +ρN1-H	1728	52	1722	v64	48	νC5=C6 + ρC6-H + ρN1-H	1723	73

Table S8. NPA charge and charge transfer in the most stable T-4H_2O-Br^--1, T-3H_2O-$[C_nmim]^+$-Br^--1, T-2H_2O-$[C_nmim]^+$-2Br^--1, and T-1H_2O-2$[C_nmim]^+$-2Br^--1 micro-hydrates with respect to the corresponding isolated fragment, respectively (in a.u.).

	T	$[C_{nmim}]^+$	Br^-	H_2O
**Monomer**	0.000	1.000	-1.000	0.000
**T-4H_2O-Br^--1**	-0.041	-	-0.929	-0.030
**T-3H_2O-$[C_2mim]^+$-Br^--1**	-0.028	0.965	-0.931	-0.005
**T-3H_2O-$[C_4mim]^+$-Br^--1**	-0.025	0.963	-0.932	-0.005
**T-3H_2O-$[C_6mim]^+$-Br^--1**	-0.025	0.963	-0.932	-0.006
**T-3H_2O-$[C_8mim]^+$-Br^--1**	-0.027	0.963	-0.931	-0.005
**T-3H_2O-$[C_{10}mim]^+$-Br^--1**	-0.027	0.963	-0.931	-0.005
**T-2H_2O-$[C_2mim]^+$-2Br^--1**	-0.046	0.957	-1.858	-0.053
**T-2H_2O-$[C_4mim]^+$-2Br^--1**	-0.044	0.955	-1.859	-0.052
**T-2H_2O-$[C_6mim]^+$-2Br^--1**	-0.044	0.955	-1.859	-0.052
**T-2H_2O-$[C_8mim]^+$-2Br^--1**	-0.046	0.955	-1.858	-0.052
**T-2H_2O-$[C_{10}mim]^+$-2Br^--1**	-0.044	0.955	-1.859	-0.052
**T-1H_2O-2$[C_2mim]^+$-2Br^--1**	-0.080	1.967	-1.862	-0.025
**T-1H_2O-2$[C_4mim]^+$-2Br^--1**	-0.077	1.965	-1.863	-0.025
**T-1H_2O-2$[C_6mim]^+$-2Br^--1**	-0.077	1.964	-1.863	-0.025
**T-1H_2O-2$[C_8mim]^+$-2Br^--1**	-0.078	1.966	-1.863	-0.025
**T-1H_2O-2$[C_{10}mim]^+$-2Br^--1**	-0.077	1.965	-1.863	-0.025
